# Single Turnover Autophosphorylation Cycle of the PKA RIIβ Holoenzyme

**DOI:** 10.1371/journal.pbio.1002192

**Published:** 2015-07-09

**Authors:** Ping Zhang, Matthias J. Knape, Lalima G. Ahuja, Malik M. Keshwani, Charles C. King, Mira Sastri, Friedrich W. Herberg, Susan S. Taylor

**Affiliations:** 1 Department of Pharmacology, University of California at San Diego, La Jolla, California, United States of America; 2 Department of Biochemistry, University of Kassel, Kassel, Germany; 3 Department of Chemistry and Biochemistry, University of California at San Diego, La Jolla, California, United States of America; 4 Department of Pediatrics, University of California at San Diego, La Jolla, California, United States of America; Cornell University, UNITED STATES

## Abstract

To provide tight spatiotemporal signaling control, the cyclic adenosine monophosphate (cAMP)-dependent protein kinase (PKA) holoenzyme typically nucleates a macromolecular complex or a “PKA signalosome.” Using the RIIβ holoenzyme as a prototype, we show how autophosphorylation/dephosphorylation of the RIIβ subunit, as well as cAMP and metal ions, contribute to the dynamics of PKA signaling. While we showed previously that the RIIβ holoenzyme could undergo a single turnover autophosphorylation with adenosine triphosphate and magnesium (MgATP) and trap both products in the crystal lattice, we asked here whether calcium could trap an ATP:RIIβ holoenzyme since the RIIβ holoenzyme is located close to ion channels. The 2.8Å structure of an RIIβ^p^_2_:C_2_:(Ca_2_ADP)_2_ holoenzyme, supported by biochemical and biophysical data, reveals a trapped single phosphorylation event similar to MgATP. Thus, calcium can mediate a single turnover event with either ATP or adenosine-5'-(β,γ-imido)triphosphate (AMP-PNP), even though it cannot support steady-state catalysis efficiently. The holoenzyme serves as a “product trap” because of the slow off-rate of the pRIIβ subunit, which is controlled by cAMP, not by phosphorylation of the inhibitor site. By quantitatively defining the RIIβ signaling cycle, we show that release of pRIIβ in the presence of cAMP is reduced by calcium, whereas autophosphorylation at the phosphorylation site (P-site) inhibits holoenzyme reassociation with the catalytic subunit. Adding a single phosphoryl group to the preformed RIIβ holoenzyme thus creates a signaling cycle in which phosphatases become an essential partner. This previously unappreciated molecular mechanism is an integral part of PKA signaling for type II holoenzymes.

## Abstract 2

The dissociation of inhibitory subunits from the prototypical cAMP-dependent protein kinase PKA is sensitive to calcium, raising the possibility that PKA's close association with calcium channels may represent a new link between calcium and cAMP signaling pathways.

## Author Summary

Kinases are important regulators in many cellular signaling processes. They bind to and phosphorylate different substrates to affect distinct signaling events. Usually, a kinase releases its substrate quickly after phosphorylation to ensure fast signaling. However, we find that this is not always true for the prototypical cAMP-dependent protein kinase PKA. PKA activity is inhibited by binding regulatory subunits, some of which are themselves PKA substrates. We show that type II regulatory subunits, such as RIIβ, are phosphorylated but do not release the kinase. These holoenzyme complexes consisting of PKA catalytic subunits and type II regulatory subunits are product traps. cAMP can bind this holoenzyme, facilitating the dissociation of the complex and unleashing PKA activity. We show that this dissociation event is modulatable by different divalent metal ions (magnesium and calcium). Since PKA is tethered to and regulates calcium channels, we propose a new link between cAMP and calcium signaling pathways—two of the major signaling molecules in the cell. We also found that this inhibitory phosphorylation event impacts the reassociation of PKA complexes. Thus, for fast and tight regulation of PKA, other signaling enzymes, such as phosphatases, are needed to remove this modification.

## Introduction

Cyclic adenosine monophosphate (cAMP)-dependent protein kinase (PKA) is a serine/threonine (Ser/Thr) protein kinase that regulates various biochemical processes including metabolism, memory, growth, and cellular differentiation [1]. In the absence of cAMP, PKA in cells exists as a holoenzyme complex consisting of a regulatory (R) subunit dimer and two catalytic (C) subunits. At low basal cAMP levels, the R_2_C_2_ holoenzyme is inactive, whereas upon hormonal stimulation, cAMP levels increase and allosteric binding of cAMP to the R subunit in the holoenzyme causes a conformational change that unleashes the catalytic activity [2–4]. PKA was the first protein kinase to be crystallized [5,6], and the C-subunit has been extensively studied as a model system for biochemical and kinetic analysis of the protein kinase superfamily [4]. Most of the states in the PKA catalytic cycle have also been captured in a crystal lattice. Divalent metal ions are required for physiological activity, and it is reported that magnesium, manganese, and cobalt can facilitate phosphoryl transfer under steady-state conditions but calcium, zinc, and others cannot [7].

While we have learned much about the kinetic properties of protein kinases from the isolated C-subunit, in cells the PKA C-subunit is typically associated with and inhibited by R-subunits that are the major receptors for cAMP in eukaryotic cells. There are four functionally nonredundant R-subunits (RIα, RIβ, RIIα and RIIβ), and isoform diversity is a primary mechanism for achieving PKA signaling specificity [4]. While both PKA RI and RII are ubiquitously expressed in mammalian cells, RI is primarily cytoplasmic and more sensitive to cAMP, whereas RII is associated with particulate subcellular fractions. RIIβ, in particular, is the predominant isoform in brain, liver, and brown and white adipose tissue [8–10]. Mice lacking RIIβ have a lean phenotype and do not become obese or insulin resistant when fed on a high-fat diet [10–12]. All R-subunits have an N-terminal dimerization/ docking (D/D) domain, a disordered linker region harboring an inhibitor sequence, and two tandem cAMP binding domains (CNB-A and CNB-B). A fundamental difference between PKA R-subunits is in the inhibitor sequence that resembles a PKA substrate and lies in the active site cleft of the C-subunit in the holoenzyme. The inhibitor sites in RII subunits (RRX**S**) are PKA substrates, while in RI subunits the inhibitor sites (RRX**A**/**G**) are pseudosubstrates. RI-subunits are thus inhibitors of the C-subunit, while the RII-subunits are substrates as well as inhibitors of the C-subunit [13]. The PKA holoenzymes are typically localized to various subcellular locations via scaffolding proteins known as A kinase anchoring proteins (AKAPs). Although some AKAPs are dual specific and can bind to both RI and RII [14], a few are specific for RI [15,16]. Most AKAPs, however, bind to RII subunits with high affinity (K_D_ = 1–5 nM), and RII-subunits are mostly associated with particulate fractions [14]. AKAPs thus serve as scaffolds for nucleating macromolecular signaling complexes by simultaneously interacting with various proteins such as kinases (PKA), phosphatases, phosphodiesterases, and ion channels [14]. For example RII-specific AKAP15/18 localizes PKA RII holoenzymes to L-type calcium channels [17]. AKAP15/18 also colocalizes PKA with phospholamban and phosphodiesterases at the sarcoplasmic reticulum (SR), thereby regulating calcium reuptake [18]. Assembly of these macromolecular complexes or “signalosomes” is specific to particular AKAPs and most likely to specific R isoforms and provides a common mechanism for spatiotemporal regulation of specific PKA holoenzymes [4].

**Fig 1:**
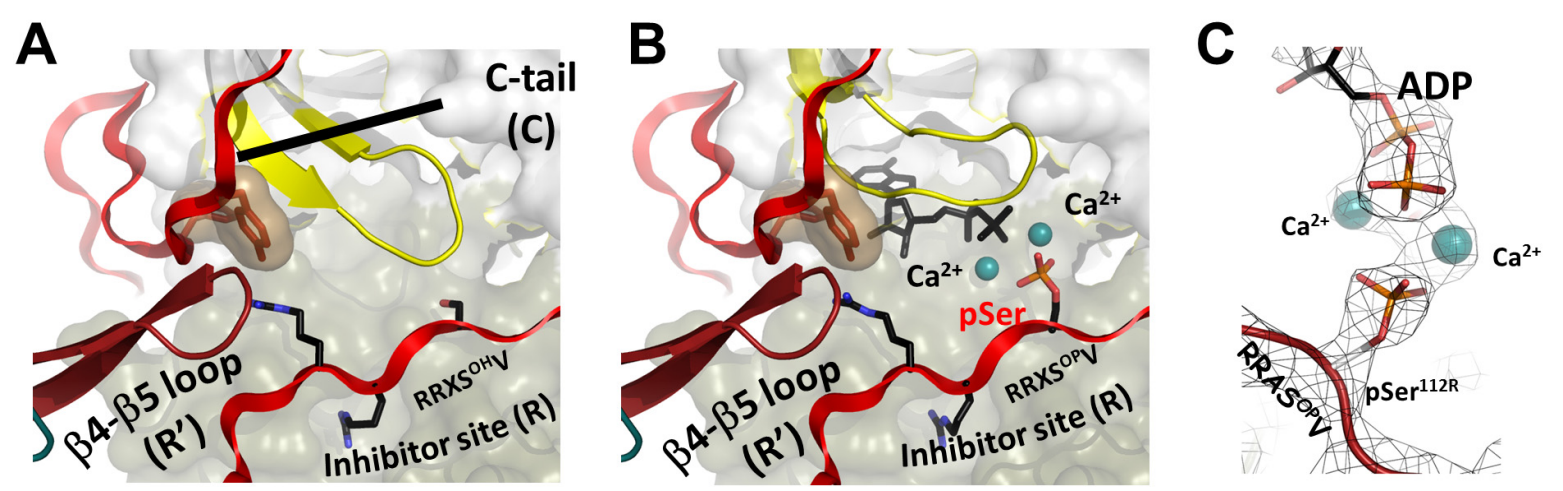
Crystal structures of the RIIβ holoenzyme showing active site phosphorylation in the presence of calcium. (A) In the apo RIIβ_2_:C_2_ holoenzyme, the β4–β5 loop from one heterodimer pushes onto the C-tail of the opposite dimer, forcing the C-subunit into a closed conformation [19]. (B) By diffusing adenosine triphosphate and calcium (CaATP) into the crystals, we trapped ADP, pRIIβ, and two Ca^2+^ ions. (C) Reaction products trapped in the crystal.

The elucidation of full-length R_2_C_2_ PKA holoenzymes structures [19,20] was a major advance that greatly enhanced our molecular understanding of PKA signaling and allosteric activation by cAMP [21]. The compact RIIβ holoenzyme], our highest resolution structure so far, provides us with an excellent model for studying type II PKA signaling. The full-length nonphosphorylated holoenzyme (Fig 1) showed surprisingly that the C-subunit was in a fully closed conformation because the β4–β5 loop of the neighboring RIIβ’plus two Mg^2+^ ions. This was the first time that both products had been trapped in a crystal lattice in any kinase. This single turnover event in which release of the phosphorylated product is controlled by cAMP, not by the transfer of the phosphoryl group, provides a unique opportunity to explore a highly relevant physiological phosphorylation event that takes place under non-steady-state conditions.

Since RII holoenzymes are often localized in close proximity to calcium channels [17], first we added CaATP to the apo RIIβ holoenzyme crystals, expecting to find a trapped transition-state mimic with ATP because calcium was thought not to support catalysis [7,24]. Instead, to our surprise, we found that we had again trapped both products which correspond to a single turnover autophosphorylation. Using rapid quench methods, we showed the phosphoryl transfer with CaATP was slower than with MgATP. Calcium could indeed support transfer of the γ-phosphoryl group either in the crystals or in solution even when adenosine-5'-(β,γ-imido)triphosphate (AMP-PNP) was used instead of ATP. This led us to reinvestigate how autophosphorylation of the RIIβ-subunit contributes specifically to the activation/inactivation cycle of the RIIβ holoenzyme and whether there is a difference between calcium and magnesium since both can support transfer of the phosphate. We examined steady-state kinetics using RIIβ bound to cAMP as a physiological protein substrate. We found that magnesium supports steady-state catalysis but calcium does not, even though it supported the phosphoryl transfer in the single turnover autophosphorylation. We then asked whether calcium and magnesium have different effects on RIIβ holoenzyme dissociation/reassociation using surface plasmon resonance (SPR). We showed that calcium was more effective than magnesium in preventing release of the phosphorylated RIIβ subunit. In contrast, the association rate of pRIIβ with C-subunit in the presence of ATP was reduced by almost 60-fold. These findings, plus our demonstration that in resting cells the RIIβ holoenzyme is fully phosphorylated, provide a more comprehensive and quantitative understanding of the role that autophosphorylation and metal ions can play in the regulation of PKA signaling for type II holoenzymes. They also describe the RIIβ holoenzyme as a single turnover system, a concept that has not been previously fully appreciated. Our model also suggests that phosphatases and phosphodiesterases are likely to be important in this cycle. This single turnover system will likely be relevant for other kinase-regulated signaling complexes.

## Results

### Crystal Structure of RIIβ Holoenzyme Soaked with Calcium and ATP

Given that RII holoenzymes are often localized in close proximity to ion channels or to mitochondria, we specifically asked whether calcium could play a role in the regulation of the PKA holoenzyme. To ascertain whether calcium could substitute for magnesium in the autophosphorylation of the RIIβ holoenzyme or whether it trapped an ATP:Ca complex, we diffused CaATP into the apo nucleotide-free RIIβ_2_:C_2_ holoenzyme crystals (Fig 1). Our expectations were that we would trap ATP and Ca^2+^ in a transition-state complex since calcium was not thought to support catalysis [7,24]. The RIIβ(1–416, R230K)_2_:C_2_ tetrameric enzyme was crystallized and then soaked overnight in 5 mM CaCl_2_ and 1 mM ATP. The structure of the full-length RIIβ_2_:C_2_ holoenzyme (S1 Table and S2 Fig), solved to a resolution of 2.8 Å, contained Ca_2_ADP bound to the active site cleft, indicating not only that Ca_2_ATP had bound but also that the phosphoryl group was transferred to RIIβ. Both products, Ca_2_ADP and pRIIβ, were trapped at the active site cleft. This structure is comparable to our previous structure in which we had trapped Mg_2_ADP in the crystal lattice with pRIIβ (S3 Fig). Phosphoryl transfer thus occurs in the presence of both MgCl_2_ and CaCl_2_. The γ-phosphoryl group of ATP was transferred to the phosphorylation site (P-site) Ser^112^ of the R-subunit, with two metal ions stabilizing ADP and the phosphorylated RIIβ in the active site of the C-subunit without significant local conformational changes between the Mg_2_:ADP and the Ca_2_:ADP bound structures (S3 Fig). The overall nucleotide-bound structure and the nucleotide-free apo structures are very similar, with a Cα RMSD of 0.67 Å between Mg_2_ADP bound and the apo RIIβ_2_: C_2_ structure [19], 0.65 Å between Ca_2_:ADP bound and the apo structure, and 0.46 Å between the two nucleotide-bound structures. In the Ca_2_:ADP structure, the C-subunit also stays in a closed conformation. Ordering of the C-tail and the N-lobe of the C-subunits into a closed conformation independent of ATP is one of the most striking features of the initial nucleotide-free RIIβ apo holoenzyme structure. This distinguishes the RIIβ holoenzyme from the heterodimer and from all previous structures of the C-subunit, which are only in a closed conformation when ATP and two magnesium ions are bound (S1 Fig and S3 Fig) [19]. The C-subunits in both of the apo RIIβ holoenzyme and metal nucleotide-bound structures have low temperature factors in both lobes, which reflect their closed conformation and ordered C-tails.

### Calcium Does Not Support Efficient Steady-State Catalysis for the RIIβ Subunit

**Fig 2:**
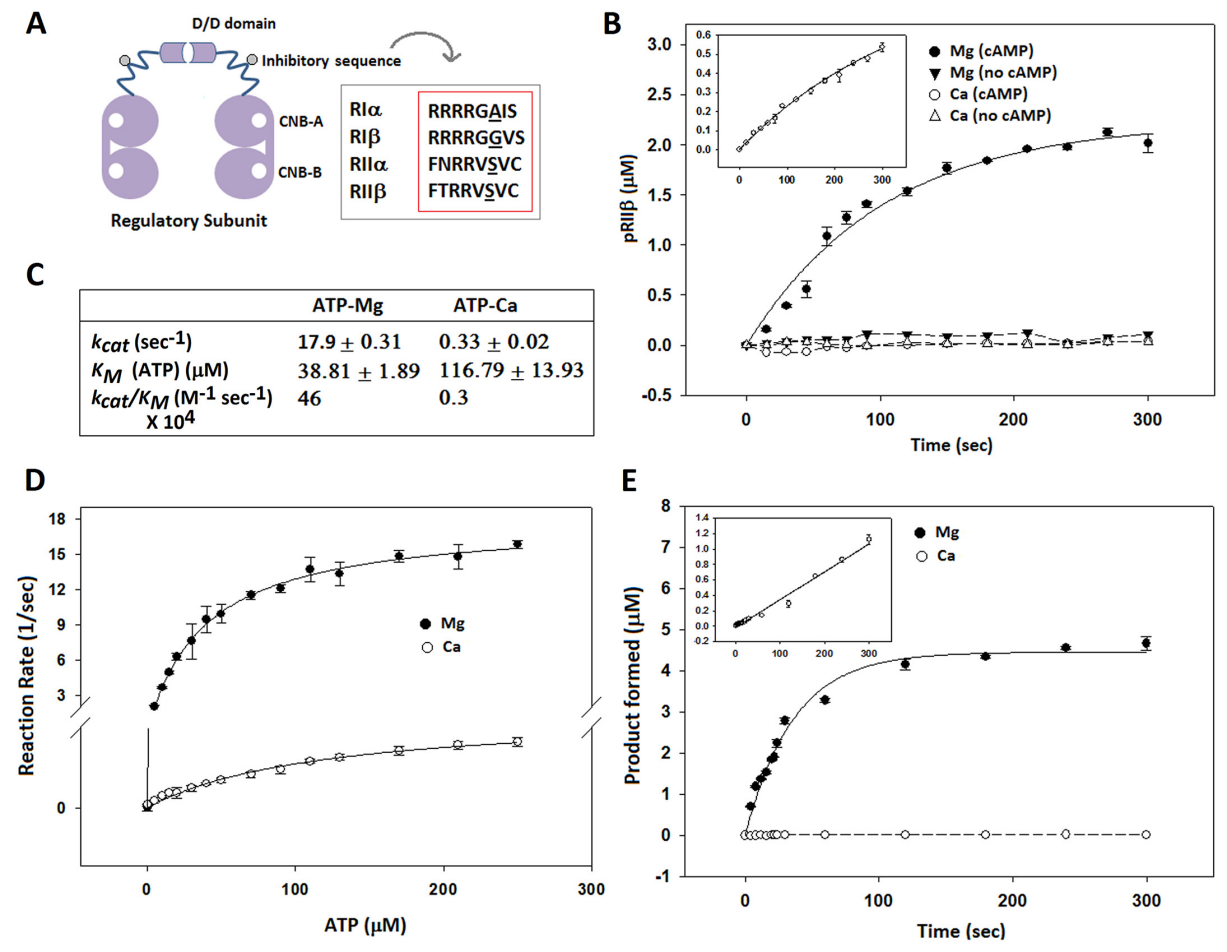
Steady-state kinetics of the catalytic subunit of PKA in the presence of Mg^2+^ versus Ca^2+^. (A) Domain organization of the four isoforms of R-subunits. The inhibitor sequence contains a pseudosubstrate site in type I R-subunits, while type II R-subunits have a substrate site. (B) Steady-state kinetics (time course) of phosphoryl transfer on the substrate site of RIIβ (40 μM) by C-subunit in the presence of Mg^2+^ and Ca^2+^. Reactions were carried out both in the presence and absence of cAMP. C-subunit was first used at 5 nM for both MgATP and CaATP. However, because of the low efficiency of the C-subunit in the presence of calcium, the measurements fell in the range of low accuracy of the scintillation counter. Hence, 50-fold higher C-subunit concentration (250 nM) was used to measure phosphorylation in the presence of CaATP and cAMP (inset) to obtain accurate results. (C) Steady-state kinetic parameters obtained for phosphoryl transfer to the peptide Kemptide in the presence of Mg^2+^ versus Ca^2+^. (D) and (E) Time course and Michealis-Menten curves for phosphoryl transfer using Kemptide as substrate. Inset shows Kemptide phosphorylation in the presence of 50-fold more C-subunit with CaATP (as compared to MgATP). The data used to make this figure can be found in S1 Data.

Having shown that calcium can support a single turnover event, i.e., autophosphorylation of the holoenzyme, we next investigated steady-state kinetics of phosphorylation using RIIβ as a physiological protein substrate. For steady-state kinetics, we used an in vitro kinase assay and radio-labelled ATP (^32^P). To measure steady-state kinetics for phosphorylation of the RIIβ subunit, we used the cAMP-bound RIIβ subunit that does not form the holoenzyme. We also used Kemptide (a LRRASLG peptide derived from the physiological PKA substrate liver pyruvate kinase [25]) as a positive reference substrate. To measure detectable phosphorylation of either Kemptide or cAMP-bound RIIβ, we needed to use 50-fold more C-subunit, confirming earlier reports in which authors could not see efficient steady-state catalysis in the presence of calcium [26]. Under these conditions, we were able to measure the kinetic parameters in the presence of calcium. The catalytic efficiency (*k*_*cat*_ */K*_*M*_) of the C-subunit to carry out steady-state phosphoryl transfer was compromised in the presence of calcium by 150-fold compared to magnesium (Fig 2). The *K*_*M*_ for ATP was increased by 3-fold in the presence of calcium, and the *k*_*cat*_ was lowered by 50-fold. No steady-state kinetic rates were detectible for the phosphorylation of RIIβ in the absence of cAMP (Fig 2B), and this is true for both MgATP and CaATP. Releasing of RIIβ is controlled by cAMP, not by the transfer of the phosphate.

We used Kemptide (a LRRASLG peptide derived from the physiological PKA substrate liver pyruvate kinase [25]), as a positive reference substrate. To measure steady-state kinetics for phosphorylation of the RIIβ subunit, we used the cAMP-bound RIIβ-subunit. The catalytic efficiency of the C-subunit to carry out steady-state phosphoryl transfer in the presence of calcium under standard conditions was undetectable for both cAMP-bound RIIβ (Fig 2B) and for Kemptide (Fig 2E), confirming earlier reports that calcium cannot support efficient steady-state catalysis [26]. Fifty-fold more C-subunit was required to obtain measurable RIIβ phosphorylation in the presence of calcium and cAMP (Fig 2B). No steady-state kinetic rates were detectible for the phosphorylation of RIIβ in the absence of cAMP, and this is true for both MgATP and CaATP. Releasing of RIIβ is controlled by cAMP, not by the transfer of the phosphate.

### RIIβ Holoenzyme Mediates a Single Turnover Event and Serves as a “Product Trap” for Both ADP and pRIIβ

**Fig 3:**
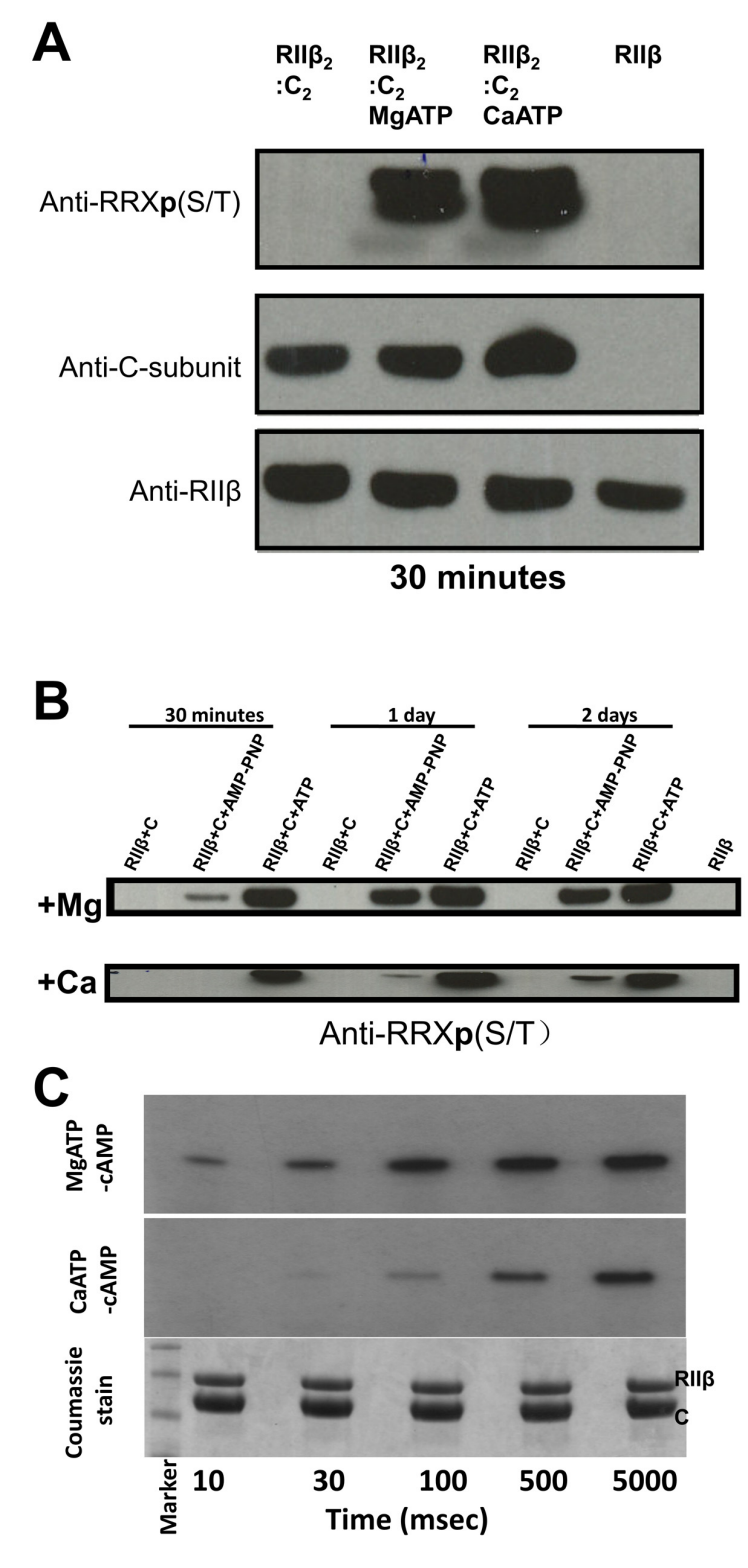
Single turnover and pre-steady-state kinetics for RIIβ holoenzyme phosphorylation in the presence of Mg^2+^ versus Ca^2+^. (A) Holoenzyme phosphorylation can be followed by gel electrophoresis in which the pRIIβ is detected by antibodies. The C-subunit phosphorylates RIIβ using MgATP or CaATP in 30 min. Shown is a representative western blot in which the C-subunit was incubated with RIIβ alone, with RIIβ and MgATP, or with RIIβ and CaATP. The starting RIIβ sample was loaded on the last lane showing no phosphorylation before incubation with C-subunit and nucleotide. Also, no phosphorylation was observed when the C-subunit and RIIβ were incubated without nucleotide, suggesting that it is not ATP contamination of either protein sample that contributes to RIIβ phosphorylation. (B) Shown is a western blot in which the C-subunit was incubated with RIIβ alone, with RIIβ and AMP-PNP, or with RIIβ and ATP. Mg^2+^ or Ca^2+^ were added to the reaction buffer, which contains nucleotides. The phosphorylation status of RIIβ was monitored at 30 min, 1 d, and 2 d. The sample with AMP-PNP shows phosphorylation of RIIβ that increases over time. (C) Pre-steady-state kinetics of RIIβ holoenzyme in the presence of Mg^2+^ and Ca^2+^. Holoenzyme phosphorylation as followed by rapid quench analysis.

Having demonstrated phosphoryl transfer in the RIIβ holoenzyme crystals, we next investigated more quantitatively the effect of metal ions on phosphorylation within the holoenzyme in solution using single turnover experiments (Fig 3). In the inactive PKA RIIβ holoenzyme, there is a 1:1 ratio of enzyme and substrate, and the S^112^ P-site of the RIIβ dimer, based on the crystal structure, is localized at the active site of the C-subunit, poised to accept the γ-phosphoryl group of ATP. We initially incubated the holoenzyme with metal ions and ATP. As shown in Fig 3A, both magnesium and calcium facilitated phosphoryl transfer to S^112^ in RIIβ very efficiently within 30 min. Since we showed previously that in the presence of MgATP and even with the generally nonreactive analogue of ATP, AMP-PNP, the C-subunit could transfer the phosphoryl group and then trap ADP and a phosphorylated form of PKA substrate peptide SP20 [27], we carried out experiments to see if the RIIβ holoenzyme could do the same. As shown in Fig 3B, the C-subunit trapped in the RIIβ holoenzyme can transfer the phosphoryl group from both AMP-PNP, although the rate of transfer for AMP-PNP is much slower than for ATP. Calcium further slows, but does not prevent, the rate of phosphoryl transfer in the presence of AMP-PNP.

**Fig 4:**
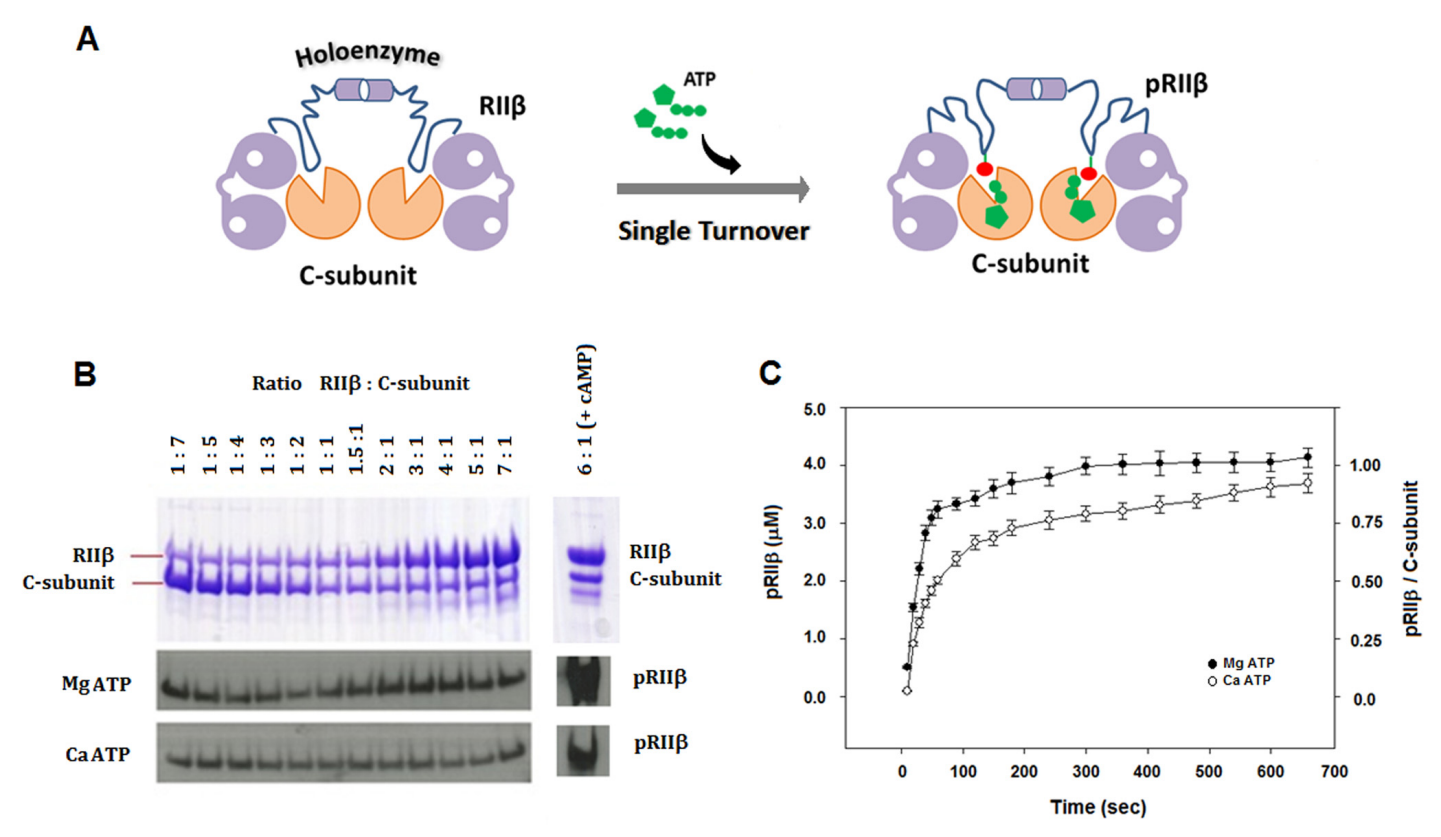
Single turnover condition for phosphoryl transfer is achieved in the PKA RIIβ holoenzyme in the presence of Mg^2+^ and Ca^2+^. (A) Formation of the RIIβ_2_:C_2_ holoenzymes leads to a special single turnover condition such that every catalytic subunit phosphorylates a stoichiometric 1:1 molecule of RIIβ. (B) Representative western blot for the single turnover seen in the RIIβ:C complex, with increasing ratios of RIIβ:C-subunit (in the absence of cAMP). Both Mg^2+^ and Ca^2+^ support the single turnover phosphoryl transfer as the holoenzymes forms at a 1:1 ratio of RIIβ:C-subunit. Addition of cAMP (1 mM) breaks the holoenzyme, and hence, the single turnover condition is lost. Band intensities for the western blots have been quantified using ImageJ software (see S4 Fig). (C) Comparison of single turnover seen in the RIIβ_2_:C_2_ holoenzyme as a function of time for MgATP versus CaATP as monitored by radio-labelled P^32^-ATP as a function of time. Both Mg and Ca support the single turnover condition, although calcium is slower than Mg in its phosphoryl transfer. The data used to make this figure can be found in S1 Data.

To monitor the rates of phosphoryl transfer in the RIIβ holoenzyme at a millisecond (ms) time scale, a rapid quench method [28] using radio-labelled γP^32^-ATP was used. As shown in Fig 3C, in the presence of MgATP, detectable amounts of pRIIβ can be seen as early as 10 ms. Phosphoryl transfer in the presence of CaATP is slower when compared to MgATP but easily detectable after 100 ms. Thus, although CaATP was not capable of efficient steady-state catalysis for the cAMP-bound RIIβ subunit as shown in the steady-state experiments above, calcium can facilitate phosphoryl transfer to the RIIβ holoenzyme in solution under single turnover conditions (Fig 3). Under these conditions, neither the ADP nor the phosphorylated pRIIβ are released from the active site; instead, the holoenzyme serves as a “product trap.” To further confirm this, we added increasing ratios of RIIβ:C-subunit and incubated the solution with MgATP and CaATP (Fig 4 and S4 Fig). As can be seen in Fig 4B, the amount of RIIβ phosphorylated was proportional to the stoichiometric concentration of C-subunit present in the reaction at 1:1. Increasing the ratio of RIIβ:C-subunit had no effect, indicating that a 1:1 complex created a single turnover condition for phosphorylation and that the product is not efficiently released. Only in the presence of cAMP was the excess of RIIβ available for phosphorylation (Fig 4B). The quantitative radio-labelled ATP measurements showed that calcium is capable of single turnover kinetics comparable to magnesium in about 10 min (Fig 4C). The RIIβ holoenzyme thus serves as a prototype for a single turnover cycle in which autophosphorylation can occur with Ca^2+^ or Mg^2+^ as the counter ions. The release of the phosphorylated RIIβ subunit is controlled by cAMP, not by the transfer of the phosphate. Instead, the RIIβ holoenzyme is an efficient product trap.

### Effects of Calcium and Magnesium on RIIβ and C-Subunit Dissociation and Association

**Fig 5:**
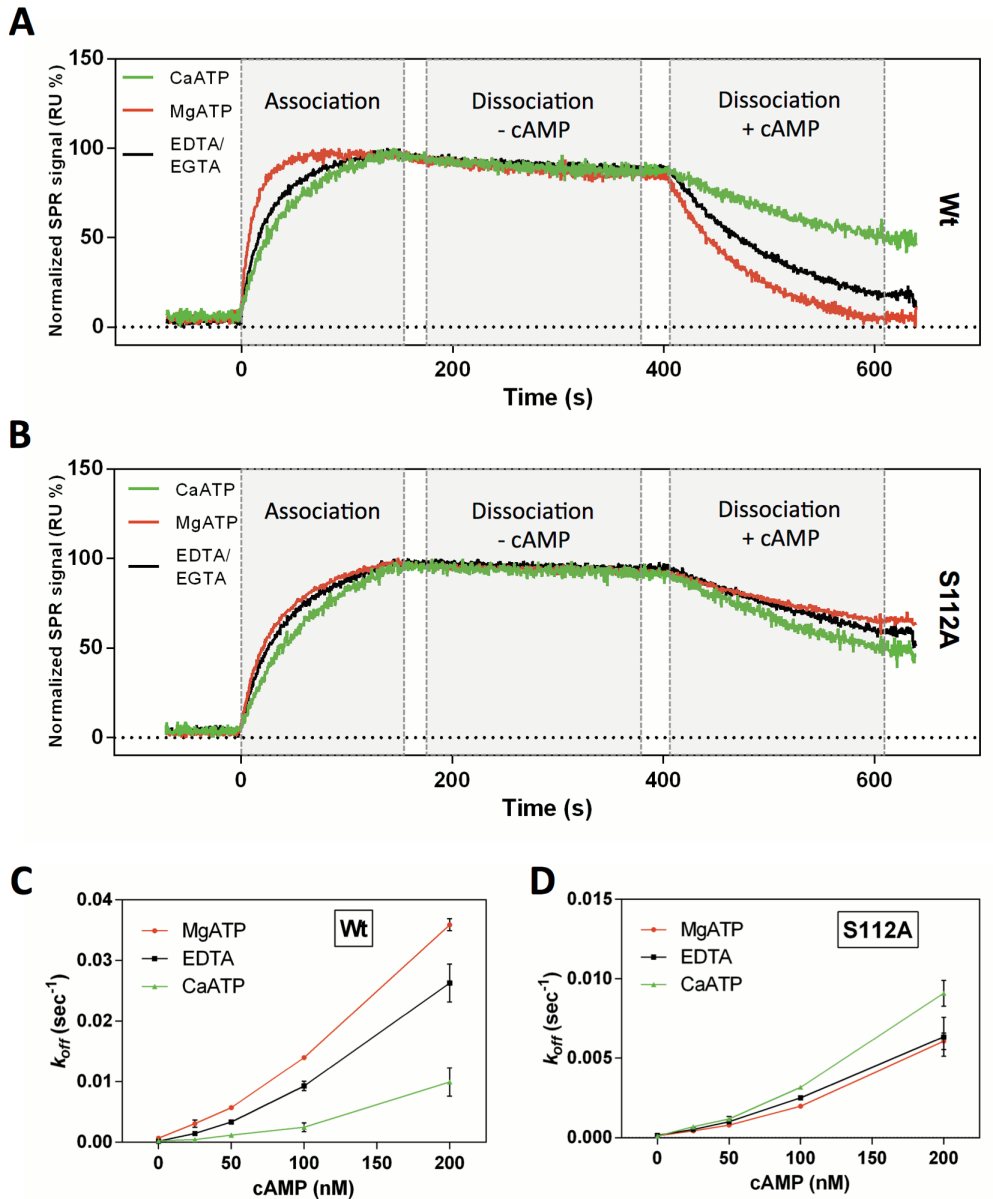
Comparison of RIIβ:C heterodimer formation and dissociation in the presence of MgATP, CaATP, or EDTA/EGTA. (A) Injection of 30 nM C-subunit over immobilized GST-RIIβ 102–416 showed complex formation under all conditions tested. The low dissociation phase (without cAMP) did not change significantly whether 10 mM Mg^2+^ or Ca^2+^ were used as cofactors, although Mg^2+^ seems to enhance the association of the complex. Holoenzyme dissociation was then induced by the injection of 100 nM cAMP in the respective buffer, resulting in a slower off-rate for holoenzyme complexes formed in the presence of Ca^2+^ compared to Mg^2+^. (B) The opposite effect can be observed for the P-site mutant GST-RIIβ 102–416 S112A, in which calcium enhances holoenzyme dissociation upon cAMP binding. Thus, the trapped phosphoryl group in combination with calcium is responsible for the decelerated dissociation. Moreover, both effects are further enhanced with increasing concentrations of cAMP for wild type (C) as well as for the S112A mutant (D). Error bars represent the standard deviation (SD) of two independent measurements. The data used to make this figure can be found in S1 Data.

Do metals play a role in RIIβ and C-subunit dissociation and association? Surface plasmon resonance was utilized to quantitatively examine the effects of magnesium and calcium on the association and dissociation of the RIIβ and C complex. For this, a deletion mutant of RIIβ, glutathione S-transferase (GST) tagged RIIβ 102–416, lacking the dimerization domain was generated for two reasons: (1) to reduce multiple binding effects that would lead to biphasic association phases making rate constant determination difficult and (2) to allow for directed immobilization on a sensor chip functionalized with an anti-GST antibody. GST-RIIβ 102–416, comprising the inhibitor site (including the phospho-acceptor serine) as well as CNB domains A and B, showed efficient holoenzyme formation in the presence of 1 mM ATP with 10 mM Mg^2+^ as well as with 10 mM Ca^2+^ (Fig 5A). In the absence of cAMP, the dissociation rate of the holoenzyme complex was extremely slow (*k*_*off*_ ~ 10^−4^ sec^-1^) and in the same range for Mg^2+^ and Ca^2+^. However, inducing holoenzyme dissociation by injecting buffer containing 100 nM cAMP showed decelerated off-rates for complexes formed in the presence of CaATP compared to MgATP (Fig 5A). The dissociation rate of the GST-RIIβ and C subunits with varying concentrations of cAMP from 25 to 200 nM is shown in Fig 5C and further confirmed that holoenzyme dissociation is slower in the presence of calcium. Representative plots for the cAMP-induced holoenzyme dissociation in the presence of ethyleneglycoltetraacetic acid and ethylenediaminetetraacetic acid (EGTA/EDTA) are shown in S5 Fig. With increasing concentrations of cAMP, this reduction of the dissociation rate was further enhanced, indicating that Ca^2+^ is able to stabilize the inhibitor site binding, thus resulting in a reduced cAMP sensitivity for the holoenzyme complex (Fig 5C).

### The Transferred Phosphoryl Group Is Responsible for Reduced Holoenzyme Dissociation with Calcium

Having shown that calcium slows down cAMP-induced dissociation, we aimed to clarify whether calcium itself or calcium in combination with the transferred phosphoryl group slows dissociation. For this, we generated a pseudosubstrate version of RIIβ in which Ser112 was replaced with alanine (Ala) (GST-RIIβ(S112A, 102–416)). This mutant was bound to a SPR sensor chip, and binding of C-subunit was determined side by side with the wild-type protein. Mutation of Ser112 to Ala barely affected binding kinetics (as determined by SPR, Fig 5B). However, contrary to wild-type RIIβ, cAMP-induced dissociation of the RIIβ(S112A):C complex was actually faster in the presence of calcium compared with magnesium (Fig 5D). Thus, the trapped phosphoryl group in combination with calcium slows dissociation in the case of the wild-type protein.

### Reassociation of Holoenzyme Is Reduced for the Phosphorylated pRIIβ Compared to the Unphosphorylated RIIβ

**Fig 6:**
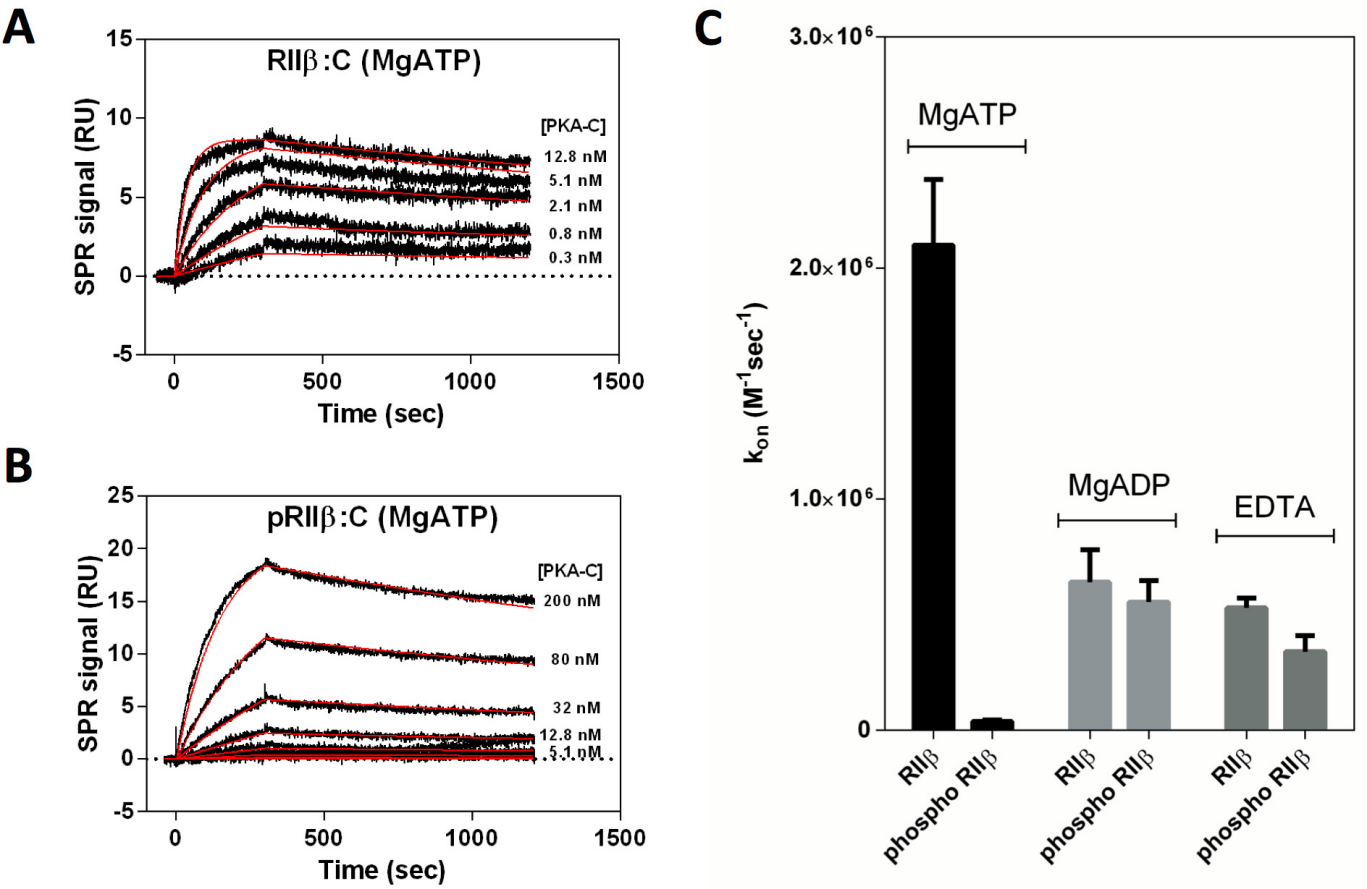
Influence of the inhibitor site phosphorylation on reassociation of the RIIβ holoenzyme. SPR was used to monitor holoenzyme formation (RIIβ:C) and reassociation (pRIIβ:C) in the presence of 10 mM MgCl_2_ and 1 mM ATP. (A) Binding of PKA-C to immobilized nonphosphorylated GST-RIIβ 102–416 resulted in strong binding with extremely low off-rates, indicating a highly stable complex. (B) For the analysis of the reassociation of phosphorylated RIIβ with C-subunit, preformed holoenzyme (pRIIβ:C:MgADP) was immobilized on the chip surface, and the catalytic subunit was dissociated by the injection of 500 nM cGMP. cGMP instead of cAMP was used to easily release the cyclic nucleotide after holo-dissociation. cAMP has a much higher affinity to the cyclic nucleotide binding sites and cannot be removed even after extensive dialysis. After removal of cGMP, the reassociation of the holoenzyme was monitored again in the presence of 10 mM MgCl_2_ and 1 mM ATP. As can be seen in (C), association of the catalytic subunit was slowed down by a factor of almost 60 compared with unphosphorylated RIIβ. However, repeating the experiments in the absence of nucleotides/metals (EDTA) and in the presence of 10 mM MgCl_2_ and 1 mM ADP revealed only slight differences in *k*_*on*_. The data used to make this figure can be found in S1 Data.

A fundamental difference between PKA R-subunits is that RII subunits are PKA substrates, while RI subunits are pseudosubstrates. After cAMP binding and subsequent dissociation of the holoenzyme complex, reassociation is thought to be unlikely without previous dephosphorylation of the RII subunit [26]. Although this was predicted in Rosen’s early work [26], it has not been addressed quantitatively. We utilized SPR to explore whether the phosphoryl group alone or the charge repulsion between the phosphorylated pRIIβ and the metal:ATP-bound C-subunit influences reassociation of the holoenzyme complex. For this, holoenzyme (GST-RIIβ(102–416):C), preformed and incubated in buffer containing MgATP, was immobilized on the SPR sensor surface. After release of the C-subunit by cyclic guanosine monophosphate (cGMP), reassociation of the C-subunit with the already phosphorylated GST-tagged RIIβ(102–416) was measured either in the presence of EDTA, MgADP, or MgATP. As shown in Fig 6, reassociation was significantly reduced when MgATP was bound to the C-subunit but was only slightly affected in the case of MgADP or in the presence of EDTA (Fig 6 and Table 1). Strikingly, in the presence of MgATP, phosphorylation of Ser^112^ in the inhibitory site of the R-subunit results in an almost 60-fold decrease in *k*_*on*_, while dissociation was not affected (Fig 6C and Table 1). These results support the prediction that the phosphoryl group at the inhibitor site of the pRIIβ subunit would be in steric hindrance with the γ-phosphoryl group of ATP at the active site of the C-subunit. Charge repulsion would also certainly contribute to the reduced on-rate. We thus conclude that in living cells it would be the dephosphorylated RIIβ-subunit that preferentially reassociates with the C-subunit to form inactive holoenzyme. However, our prediction is that it would become immediately rephosphorylated upon holoenzyme reassociation. This scenario implicates an important role for phosphatases, and to further validate this hypothesis, we looked at the phosphorylation state of RIIβ in different cell types.

**Table 1:** SPR-derived rate and equilibrium binding constants of the interaction of PKA C-subunit with phospho- and nonphospho forms of RIIβ. A series dilution of PKA-C (0.3–200 nM) was injected over the phospho- and nonphospho forms of immobilized GST-RIIβ 102–416 (80–120 resonance units [RUs]) in the presence of MgATP, MgADP, and chelators (EDTA) only. BIAevalution 4.1.1 (GE Healthcare) was utilized to determine the rate constants *k*_*on*_ and *k*_*off*_ using a global fit analysis assuming a 1:1 Langmuir binding.

		MgATP	MgADP	EDTA
**RIIβ:C**				
	*k*_*on*_ [M^-1^sec^-1^]	2.1 (+/- 0.3) x 10^6^	6.4 (+/- 1.4) x 10^5^	5.3 (+/- 0.4) x 10^5^
	*k*_*off*_ [sec^-1^]	3.0 (+/- 0.6) x 10^−4^	1.5 (+/- 0.6) x 10^−4^	2.1 (+/- 0.5) x 10^−4^
	*K*_*D*_ [nM]	0.14 (+/- 0.01)	0.24 (+/- 0.15)	0.39 (+/- 0.13)
**pRIIβ:C**				
	*k*_*on*_ [M^-1^sec^-1^]	3.8 (+/- 0.7) x 10^4^	5.5 (+/- 0.9) x 10^5^	3.4 (+/- 0.7) x 10^5^
	*k*_*off*_ [sec^-1^]	2.6 (+/- 0.1) x 10^−4^	1.2 (+/- 0.3) x 10^−4^	1.1 (+/- 0.4) x 10^−4^
	*K*_*D*_ [nM]	7.0 (+/- 1.7)	0.22 (+/- 0.1)	0.31 (+/- 0.04)

Mean +/- SD

### Endogenous RIIβ Holoenzyme Is Phosphorylated in Cells and Is Dephosphorylated by Phosphatases following Stimulation by cAMP

**Fig 7:**
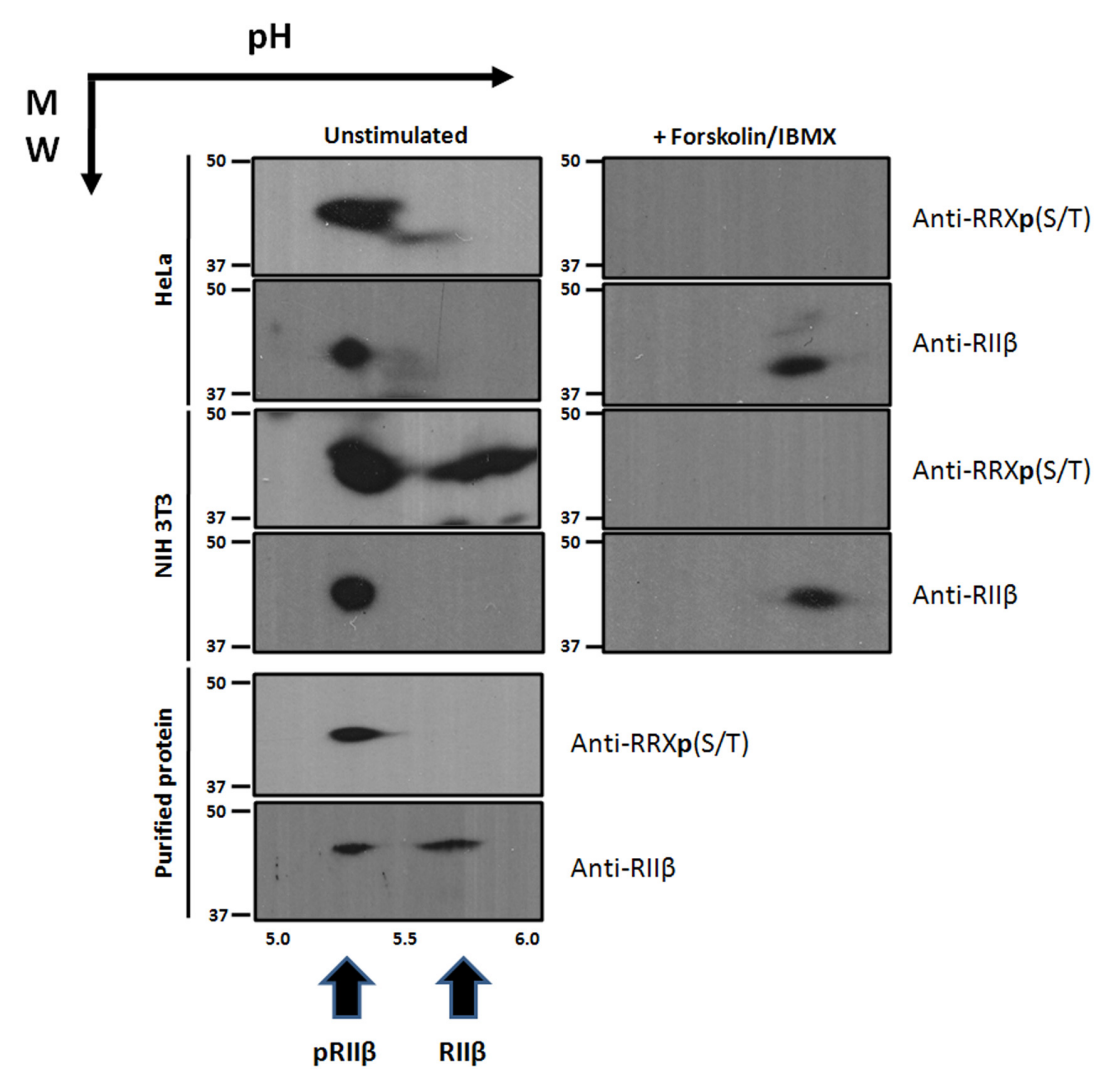
Endogenous RIIβ is constitutionally phosphorylated in 3T3 and HeLa cells. pH gradient is shown horizontally, and molecular weight (MW) gradient is shown vertically. Left panel: The purified pRIIβ and RIIβ protein mixture, prepared in 2-D electrophoresis buffer, is shown in the last lane, displaying the successful separation and relative position of pRIIβ and RIIβ in a 2-D gel. Phosphorylated pRIIβ can be recognized by both of the phospho-PKA substrate antibody (Anti-RRXp(S/T)) and the RIIβ antibody (anti-RIIβ), while unphosphorylated RIIβ is only recognized by the RIIβ antibody (anti-RIIβ). The first and second two lanes show that RIIβ exists as phosphorylated pRIIβ in both untreated HeLa and HeLa cells. In 3T3 cells, one protein near the site of pRIIβ is recognized by phospho-PKA substrate antibody (Anti-RRXp(S/T); however, it is not recognized by the RIIβ antibody. Right panel: In HeLa and 3T3 cells treated with 50 μM forskolin and 50 μM IBMX for 15 min prior to adding the lysis buffer, the PKA RIIβ becomes completely dephosphorylated.

Having quantitatively defined the effects of RIIβ phosphorylation on association/dissociation of the holoenzyme, we next asked about the phosphorylation state of RIIβ holoenzyme in cells. Based on gel filtration chromatography, the RIIβ holoenzyme does not dissociate in vitro in the presence of MgATP, which is also confirmed by the SPR data (Fig 5 and S6 Fig). These combined data suggest that the inhibitor site of RIIβ is only accessible to phosphatases once it is unleashed from the active site following binding of cAMP. Once holoenzyme forms with the dephosphorylated RIIβ subunit, it would be immediately rephosphorylated and then trapped in its phosphorylated state. To validate this prediction, two-dimensional gel electrophoresis analysis was used to explore the endogenous phosphorylation state of RIIβ in cells (Fig 7). HeLa and NIH 3T3 cells were cultured, lysed for 2-D gel analysis, and checked for the phosphorylation status of RIIβ using PKA phospho-substrate antibody and overall RIIβ antibody. As shown in Fig 7, RIIβ is completely phosphorylated in both untreated HeLa and 3T3 cells in the resting state. Similar results have been previously reported in cardiomyocytes, in which it was found that RIIβ under basal conditions is phosphorylated [29]. Once the cells are treated with forskolin, an activator of adenylyl cyclase, and 3-isobutyl-1-methylxanthine (IBMX), a general inhibitor of phosphodiesterases, for 15 min to elevate the cAMP level, RIIβ gets dephosphorylated, suggesting that after cAMP activation, the phosphorylated RIIβ can be exposed to phosphatases and be dephosphorylated, which, based on our above results, is a critical step that would greatly facilitate the reassociation of PKA RII and the C-subunit.

## Discussion

The recent explication of the structures of. full-length R_2_C_2_ PKA holoenzymes [4,19,20] made us appreciate that PKA signaling cannot be fully understood by simply looking at free regulatory and catalytic subunits. To include an understanding of the highly dynamic macromolecular PKA signaling complexes that are controlled by oscillations between kinases and phosphatases, cyclases and phosphodiesterases, and cAMP and calcium, we also need to appreciate that protein kinases have evolved to be highly sophisticated and regulated macromolecular switches, not catalytic enzymes that turnover lots of substrate. Recognition of these fundamental principles forces us to think of PKA signaling as part of a “signalosome” in which full dissociation of R- and C-subunits may not be required or relevant. Under these conditions, PKA would not be working as described by classical Michaelis-Menten kinetics but instead would function as a single turnover system in which autophosphorylation of the R-subunit can play an important role.

We provide here an example of how the turnover of a single phosphate in the RIIβ subunit (pSer^112^) introduces multiple levels for regulation of this cAMP-mediated PKA switch. Our recent X-ray crystal structure of the PKA RIIβ holoenzyme showed that both products, Mg_2_ADP and pRIIβ, could be trapped following the addition of MgATP [19]. Here we provide kinetic parameters for autophosphorylation of the RIIβ subunit in both steady-state and single turnover conditions. We also show that calcium can facilitate phosphoryl transfer in type II PKA holoenzymes in crystals and in solution, but this is restricted to a single turnover. The products are not released because dissociation for both RIIβ and pRIIβ is regulated by cAMP. The holoenzyme thus serves as a trap for both products. This discovery demonstrates that single turnover events can be an important part of cell signaling, in contrast to classic Michaelis-Menten kinetics.

Having shown that Ca^2+^ can support the single turnover of the γ-phosphoryl group of ATP in the RIIβ holoenzyme, we then asked how Ca^2+^ affects dissociation of the phosphorylated holoenzyme (pRIIβ:C). To quantitatively measure the effects of metals ions on holoenzyme association/dissociation, we used SPR. We found that in the absence of cAMP, holoenzyme dissociation is extremely slow for both Mg^2+^ and Ca^2+^; it is not significantly influenced by the type of metal ion. After addition of cAMP, however, the C-subunit is rapidly released in the presence of Mg^2+^, whereas the off-rate is decreased significantly when the counter ion is Ca^2+^ (Fig 5). The Ca^2+^ bound phosphorylated RIIβ-holoenzyme thus has a reduced sensitivity to cAMP, which was indicated previously [30] by the increase in the activation constants for RII holoenzymes formed in buffer containing Ca^2+^. However, it should be pointed out that although a significant effect of Ca^2+^ can be seen for the R:C heterodimer, this effect could be even more pronounced in the R_2_:C_2_ holoenzyme, where the active site of the catalytic subunit is further stabilized through interactions with the R-subunit from the opposite heterodimer [19,30]. This quaternary constraint, for example, leads to a 10-fold increase in the K_a_ for activation of the R_2_C_2_ holoenzyme by cAMP compared to the R:C heterodimer (65 nM versus 584 nM) [19].

**Fig 8:**
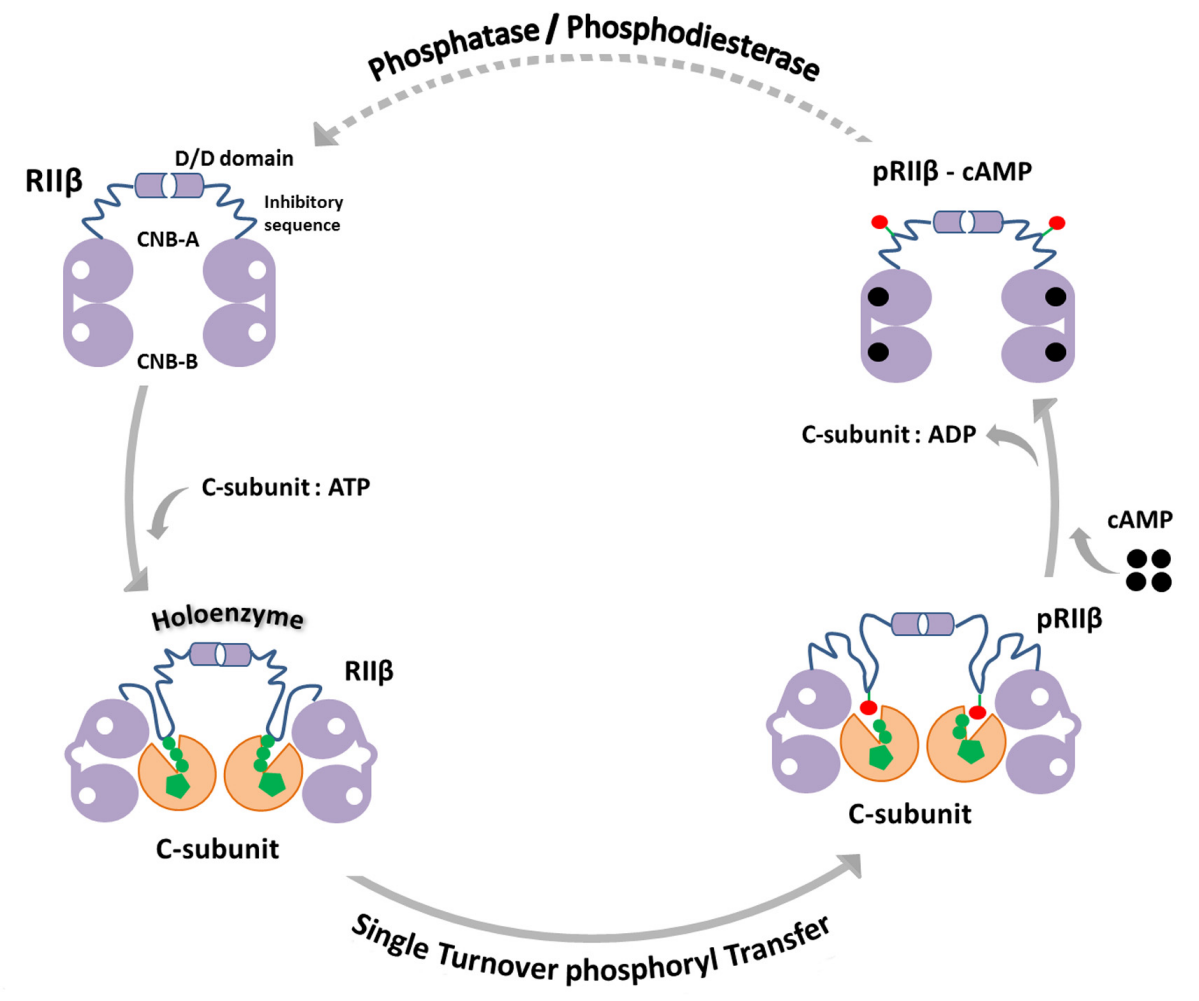
Role of single turnover phosphorylation in the regulation of PKA signaling. Type II holoenzymes of PKA are under an additional feedback regulation that is mediated by the phosphorylation of the R-subunits. Holoenzyme formation creates a single turnover condition in which the catalytic subunit phosphorylates the R-subunit. Increased cAMP levels dissociate the holoenzyme as the R-subunit binds cAMP or at least unleashes the inhibitor site from the active site cleft of the C-subunit. Reassociation of the inactive holoenzyme requires the concerted action of phosphodiesterases that hydrolyse cAMP and phosphatases that remove the phosphoryl group from the inhibitor site of RIIβ. A phosphorylated yet cAMP-free RIIβ is unable to bind efficiently to the catalytic subunit. This therefore creates a self-regulating cycle that contributes to the regulation of PKA signaling. Whether the R- and C-subunits fully dissociate under these single turnover conditions is not clear, but full dissociation is not necessary. The only necessary requirement for activation is that the inhibitor site of the RIIβ subunit be released, allowing access of the nearby tail of a channel or receptor.

To quantitate the effects of RIIβ phosphorylation on association/dissociation, we used SPR with immobilized pRIIβ and RIIβ and measured binding of C-subunit in the presence and absence of nucleotides. Our results indicate that the phosphoryl group at the inhibitor site would collide with the γ-phosphoryl group of ATP, which is bound to the active catalytic subunit. This supports the hypothesis that once phosphorylated RIIβ is released by cAMP, the phosphoryl group needs to be removed by a phosphatase prior to this point in order to optimize rapid reassociation (Fig 8). Although this was predicted previously in Ora Rosen’s early work [26], it has not been addressed in a quantitative way subsequently. Our results for the first time strongly suggest that phosphatases play an important, perhaps essential, role in the PKA activation cycle for RII holoenzymes, in addition to their obvious role in dephosphorylating other substrates. We also used 2-D gel electrophoresis to show that RIIβ in the resting state is fully phosphorylated (Fig 7) and only becomes dephosphorylated following activation with forskolin and IBMX. Recent work suggests that phosphodiesterase (PDE) can bind directly to the RIα subunit and suggests that protein-bound cAMP is the preferred substrate for PDE [31]. PDEs, as well as phosphatases, are thus also likely to be an integral part of this single turnover signaling complex.

To further understand the importance of the single phosphate, we compared RIIβ with a P-site mutant (RIIβ S112A) that cannot be phosphorylated. The results further emphasize the importance that a single phosphate at the inhibitor site can have and also how metal ions can influence the dissociation process. The mutant clarifies that calcium slows cAMP-induced dissociation due specifically to the presence of the transferred phosphoryl group. It also shows the importance that a single turnover event, mediated by autophosphorylation, can have on PKA signaling. It also gives additional rationale for colocalization of phosphatases with RII holoenzymes, as this can have an important regulatory effect on the holoenzyme itself, not just on the nearby substrate that is tethered to the complex. The trapped product state makes the holoenzyme ready for fast dissociation upon cAMP binding, but reassociation now is also influenced by the colocalized phosphatase. The colocalization of the RII subunit with calcineurin, a calcium-activated phosphatase, is a particularly significant example in which the phosphorylated RII subunit is the best-known substrate of calcineurin [4,32,33]. In this case, the kinase and the phosphatase could be both influenced by calcium. RI holoenzymes are not regulated in this way by autophosphorylation.

The studies described here define the molecular features of a dynamic macromolecular PKA RIIβ molecular switch (Fig 8). Although PKA nucleates the complex, the signaling system includes other signaling proteins such as phosphatases and PDEs. It is a “signaling system” or “PKA signalosome” that must also be localized in close proximity to dedicated substrates such as ion channels, transporters, and receptors, which is typically achieved by AKAPs. By defining in quantitative terms the RIIβ signaling cycle, we introduce an additional novel concept by showing that the autophosphorylation of RIIβ is also a significant feature of this signaling complex. Adding a single phosphate to RIIβ creates a multifaceted signaling cycle in which the phosphorylated RIIβ, metal ions, and phosphatases all play an essential role in regulating the event that was initiated by cAMP. Since this PKA signalosome is often located in close proximity to ion channels, it is possible that in this microenvironment, calcium may also play a physiologically relevant role in regulating the cycle in a variety of ways that were not previously appreciated.

## Materials and Methods

### Protein Expression and Purification

The rat regulatory subunit RIIβ was overexpressed in *Escherichia coli* and purified as described previously. As previously described [19], a major challenge for obtaining crystals of full-length tetrameric PKA holoenzymes is likely due to the dynamic nature of the different domains. We then generated two mutants of RIIβ. Specifically, we replaced the essential Arg in each phosphate binding cassette with Lys. Although the wild-type construct and the B domain mutant were set up in parallel for crystallization, the R230K mutant was the only one that yielded well-diffracting crystals. All RIIβ proteins were expressed in *E*. *coli* BL21 (DE3) (Novagen) and purified as described previously [19,23,34] using cAMP resin and cGMP elution. Site-directed mutagenesis of RIIβ Ser112 to Ala was performed using the QuickChange mutagenesis kit (Stratagene). The mouse catalytic subunit (isoform α) was overexpressed in *E*. *coli* and purified as described previously [19].

### Complex Formation and Crystallization

RIIβ subunit and wild-type C-subunit were mixed in a 1:1.2 molar ratio and spin dialyzed at 4°C into holoenzyme buffer containing 20 mM MES (pH 5.8), 50 mM NaCl, and 1 mM TCEP-HCl. The complex was then gel filtered through Superdex 200 in the same holo buffer to remove excess C-subunit. The complex protein was concentrated to approximately 12 mg/ml for crystallization. The RIIβ(R230K):C apo complex was crystallized as described previously [19]. To make CaATP binding holoenzyme crystals, the apo holoenzyme crystals were soaked with 20% PEG8000, 8% ethylene glycol, 20 mM HEPES, pH 7.5, 5 mM CaCl_2,_ and 1 mM ATP in pH 5.8 solution overnight.

### Data Collection and Processing

The best RIIβ (1–416:R230K)^p^_2_:C_2_:(Ca_2_ADP)_2_ holoenzyme crystal diffracted to 2.8 Å. This dataset was collected at Advanced Light Source beamline 8.2.2 2 and then processed using HKL2000 program package. Initial phases were obtained using the apo RIIβ holoenzyme (PDB:3TNP) as a search model by the CCP4 package program PHASER. The refinement was done using the REFMAC5 program in CCP4 and simulated annealing process in PHENIX programs. Water molecules were added to the model at the final step of refinement. The final models were evaluated by PROCHECK.

### Pre-Steady-State Kinetic Measurements

Pre-steady-state kinetic measurements were made using a KinTek model RGF-3 quench flow apparatus following a previously published procedure [28]. The apparatus consists of three syringes driven by a stepping motor. Typical experiments were performed by mixing equal volumes of the PKA RIIβ holoenzyme (2 μM) in one reaction loop and P^32^-ATP (5,00–15,00 cpm/pmol) and MgCl_2_ (10 mM) or CaCl_2_ (100 μM), respectively, in the second reaction loop—all in the presence of 100 mM Mops, pH 7.4, and 5 mM DTT. The reaction was quenched with 10% SDS in the third syringe, and the reactions were separated using 4%–12% SDS-PAGE gel as described above.

### Steady-State Kinetics

Steady-state kinetic measurements were carried out with a reaction mix containing 50 mM MOPS pH 7.0, 50 mM NaCl, 1 mM ATP, and γ^32^P radio-labelled ATP (specific activity 500–1000 cpm/pmol) in a final volume of 20 μL. The various substrates in a volume of 10 μL were added to 5 μL of the reaction mix containing a 10 mM concentration of free metal ions (MgCl_2_ or CaCl_2_), and the reaction was initiated by 5 μL of catalytic subunit. The amount of cAMP used was 1 mM. Kemptide was used at a concentration of 1 mM, and RIIβ was used at 40 μM. The reaction was quenched with 90 μL of 30% acetic acid. 50 μL of the quenched reaction was then spotted on p81 phosphocellulose paper, washed three times for 5 min each with 5% phosphoric acid and then washed once with acetone, air dried, and counted in a liquid scintillation counter.

Time course measurements were carried out for 300 s with 20 nM enzyme with magnesium and 1 μM enzyme with calcium. 50-fold more enzyme was required in the presence of calcium for measureable phosphoryl transfer activity at the same scale with magnesium. Michaelis-Menten kinetics were carried out with 35 nM enzyme, and the reactions were incubated for 10 min. All curves were fit to classical steady-state equations using the nonlinear regression fitting module of Sigma-plot software.

### Single Turnover Using pRIIβ Antibodies

The concentration of ATP used in the reaction buffer was 1 mM when the nucleotides were included. The reaction mixture contained approximately 300 nM RIIβ_2_:C_2_ holoenzyme in holoenzyme, alone or in the presence of 1 mM nucleotide (ATP or AMP-PNP) and 10 mM MgCl_2_ (or CaCl_2_) at pH 5.8. The reaction was incubated at room temperature for the first 30 min and was then kept at 4°C. The reaction were quenched at 30 min by taking samples of the reaction mixture, mixing them 1:1 with SDS-PAGE sample buffer, and heating the samples for 10 min at 70°C. These samples were analyzed by western blot with antibodies against the C-subunit (BD Biosciences), RIIβ (Biomol), and against R-R-X-phosphorylated pS/T, the PKA consensus sequence that RIIβ contains at its inhibitor site (Cell Signaling Technology).

### Single Turnover Kinetics with Increasing Ratios of RIIβ:C-Subunit

Increasing stoichiometric ratios of RIIβ:C-subunit were incubated in 50 mM MES pH 5.8, 50 mM NaCl, and 1 mM DTT. The highest ratio of RIIβ:C-subunit was incubated with cAMP (1 mM) as a control. After incubating the proteins for 15 min, 1 h and 3 h (three sets), the reaction was initiated by adding 1 mM ATP and 10 mM metal (MgCl_2_ or CaCl_2_). Phosphoryl transfer was allowed to go on for 10 min, after which the reaction was quenched by adding SDS-PAGE sample buffer and heating the samples for 10 min at 70°C. Western blots were run and the phosphorylation status of RIIβ was probed with phospho-specific antibodies as described above. All three time points showed similar results. The bands in the western blots were quantified by ImageJ software, as described previously [27].

### Single Turnover Kinetics Using γP^32^-ATP

For quantitative single turnover kinetics, 4 μM of RIIβ-holoenzyme (2 μM R_2_:C_2_) was incubated with ATP (1 mM) and γP^32^-ATP (specific activity 500–1,000 cpm) in buffer containing 50 mM MES pH 5.8 with 10 mM metal (MgCl_2_ or CaCl_2_). The reaction was quenched by mixing 10 μL of reaction mix with 90 μL of 30% acetic acid at varying time points until about 660 s (11 min). 20 μL of this mix was then spotted on a p81 phosphocellulose filter disks and washed and dried with 5% phosphoric acid and acetone. γP^32^ transferred to RIIβ was measured in a liquid scintillation counter.

### SPR Analysis of Holoenzyme Formation and Dissociation

A Biacore T100 SPR instrument (GE Healthcare) was used to acquire kinetics of the RIIβ holoenzyme formation under different buffer conditions. For this, an anti-GST antibody (Carl Roth) was coupled to a CM5 sensor chip (GE Healthcare) using standard NHS/EDC chemistry. On this functionalized chip, a cyclic nucleotide-free

GST-fusion of PKA-RIIß wild type and S112A mutant lacking the dimerization domain (RIIβ 102–416) was immobilized on parallel flow cells to a level of 80 resonance units (RUs). PKA catalytic subunit (30–0.12 nM) was then injected in buffer A (20 mM MOPS, 150 mM NaCl, 100 μM EDTA, 100 μM EGTA, 0.01% P20) alone or supplemented with 1 mM ATP and 10 mM MgCl_2_ or CaCl_2_ each at a flow rate of 30 μL/min at 25°C for 150 s or 300 s to monitor association of the holoenzyme. Dissociation was induced by switching to buffer lacking C-subunit for 150 s or 900 s. For the analysis of cAMP-induced dissociation, the respective buffer containing cAMP at several concentrations (ranging from 25 nM to 200 nM) was injected for 200 s. Regeneration of the chip surface was carried out by the injection of 10 mM Glycine (pH 1.9) for 30 s at 30 μL/min.

Analysis of the interaction of PKA catalytic subunit to already phosphorylated pRIIβ was carried out using SPR by monitoring association and dissociation in real time. For this, phosphorylated holoenzyme was obtained by mixing PKA catalytic subunit and GST- RIIβ102–416 in a molar ratio of 1.2:1 in buffer A for 10–15 min, followed by a dialysis against buffer A supplemented with 1 mM ATP and 10 mM MgCl_2_ for 1 h at RT. This holoenzyme dimer was then immobilized to a level of 120 RU on the anti-GST-chip surface, and catalytic subunit was released by the injection of 500 nM cGMP. After 100 s of buffer flow (cGMP release), reassociation of the holoenzyme was carried out by the injection of several concentrations of catalytic subunit (0.3 nM–200 nM) for 300 s in buffer A only or buffer A containing 10 mM MgCl_2_ and either 1 mM ATP or ADP. Dissociation was monitored in the respective buffer without PKA catalytic subunit for 900 s. For the interaction analysis of nonphosphorylated RIIβ with PKA catalytic subunit, GST-RIIβ 102–416 was immobilized to a level of 80 RUs on a parallel flow cell allowing side-by-side analysis. Surfaces were regenerated with 10 mM Glycin (pH 1.9) for 30 s at 30 μL/min after each cycle. Rate constants were determined by nonlinear curve fitting (global fit analysis) using the software BIAevaluation 4.1.1 or T100 Evaluation 2.0.4 (GE Healthcare).

### Cell Culture, Two-Dimensional Gel, and Western Blot

HeLa and NIH 3T3 cells were cultured in standard DMEM medium supplemented with 10% FBS. Cells were washed twice with PBS and harvested in 300 μL lysis buffer containing 9 M urea/2% CHAPS and complete protease inhibitor cocktail tablets (Roche; Indianapolis, Indiana. The forskolin and IBMX treated cells are prepared by adding 50 μM forskolin and 50 μM IBMX for 15 min prior to adding lysis buffer. Protein (100–250 μg) was diluted in 2-D electrophoresis buffer (2 M thiourea, 5 M urea, 0.25% CHAPS, 0.25% Tween-20, 0.25% SB-3, 10% isopropanol, and 12.5% water-saturated butanol) containing 15 mg DTT and 0.5% ampholytes (pI ranges 3–10 or 5–8; Bio-Rad; Hercules, California) to a final volume of 0.2 ml and passively absorbed onto immobilized pH gradient strips (pI ranges 5–8 or 3–10 NL (nonlinear); Bio-Rad) for 4 h at 20°C, followed by active rehydration (50 V at 20°C) for 8 h. Isoelectric focusing was performed for 55,000 V-h on a Protean IEF System (Bio-Rad), and electrophoresis was performed with the Criterion gel system (Bio-Rad). 2-D gels were transferred to PVDF, and western blots were performed using phospho-PKA substrate antibody (Cell Signaling) and RIIβ antibody (Biomol).

## Supporting Information

S1 Data Excel spreadsheet containing, in separate sheets, the underlying numerical data for Figs 2B–2E, 4C, 5C, 5D and 6C and S4 Fig. (XLSX)

S1 Fig C-subunit in open and closed conformations. (A) The C-subunit is shown as a space-filling model with the N-lobe residues (14–121) in white and the C-lobe residues (122–350) in tan. The C-subunit conformations are termed “open” and “closed” on the basis of the relative orientations of the N- and C-lobes with respect to each other [35]. Left: The C-subunit is in a typical open conformation without nucleotide binding to the active site (in RIIα:C heterodimer [22], PDB ID: 2QVS). The glycine-rich loop (pink) is raised. The active site is open. In the open conformation in the absence of ATP, the C-tail in this region is disordered. Middle: The C-subunit is in a typical closed conformation with nucleotide binding to the active site (in RIα:C heterodimer [23], PDB ID: 2QCS). The active site is closed with nucleotide binding. The C-tail is ordered and folded over the N-lobe. Right: The C-subunit is in a closed conformation without nucleotide binding to the active site (only seen in RIIβ holoenzyme, PDB ID: 3TNP). (B) The glycine-rich loop is raised and the active site is open in the 2QVS structure (depicted in yellow), compared to the fully closed conformation observed in the 2QCS (depicted in cyan) and 3TNP structures (depicted in grey). AMP-PNP and two Mn ions from 2QCS are colored black. (C)The dynamic nature of the C-tail. In the closed conformation, the C-terminal tail (colored olive, PDB ID: 2QCS) is folded over the N-lobe in which two residues, Phe327 and Tyr300, are an essential part of the ATP binding site. In the open conformation in the absence of ATP, the C-tail (colored gray, PDB ID: 2QVS) in this region is disordered
(TIF)

S2 Fig Stereoview of part of the RIIβP2: C_2_: (Ca_2_ADP)_2_ structure in the 2.8 Å resolution 2*F*o–*F*c map. (TIF)

S3 Fig The conformation and B factor analysis of the C-subunit in different R:C complexes. The left bottom is the scale bar for B factors.
(TIF)

S4 Fig Single turnover autophosphorylation as seen in the RIIβ holoenzyme. (A) Varying ratios of RIIβ were incubated with the C-subunit (in the absence of cAMP) with MgATP and CaATP. Phosphorylation of RIIβ was assessed by phosphor-Ser specific antibodies. Band intensities for the western blots were quantified using ImageJ software, as described previously [27]. (B) Band intensities in the presence of MgATP. (C) Band intensities in the presence of CaATP. The data used to make this figure can be found in S1 Data.
(TIF)

S5 Fig cAMP induced dissociation of the RIIβ holoenzyme. SPR was used to monitor the dissociation of the RIIβ holoenzyme (GST-RIIβ 102–416:C) induced by the injection of various concentrations of cAMP (25–200 nM). The representative plot shows holoenzyme formation with C-subunit (30 nM) and dissociation in buffer lacking ATP and metal ions (EDTA/EGTA 100 μM each).
(TIF)

S6 Fig The RIIβ holoenzyme does not dissociate in the presence of MgATP. (A) Gel filtration chromatography shows that the preformed RIIβ holoenzyme does not dissociate in MgATP solution. Buffer condition: 200 mM NaCl, 20 mM MES, 1.0 mM TCEP, 5 mM MgCl_2_ and 1 mM ATP, pH 5.8 (B) SDS-PAGE analysis of the holoenzyme peak.
(TIF)

S7 Fig SPR-based analysis of holoenzyme formation of wild-type RIIβ and S112A. SPR was utilized to acquire binding data of holoenzyme formation for wild-type RIIβ and the P-site mutant S112A. Rate constants were determined using the Biacore T100 Evaluation Software (GE Healthcare), with global fit analysis assuming a 1:1 Langmuir binding model.
(TIF)

S1 Table Data collection and refinement statistics. (DOCX)

## Abbreviations

ADP: adenosine diphosphate
AKAP: A kinase anchoring protein
Ala: alanine
AMP-PNP: adenosine-5'-(β,γ-imido)triphosphate
ATP: adenosine triphosphate
CaATP: adenosine triphosphate and calcium
C-subunit: catalytic subunit of PKA
cAMP: cyclic adenosine monophosphate
cGMP: cyclic guanosine monophosphate
D/D: dimerization/docking
CNB: cAMP binding domain
EDTA: ethylenediaminetetraacetic acid
EGTA: ethyleneglycoltetraacetic acid
GST: glutathione S-transferase
IBMX: 3-isobutyl-1-methylxanthine
MgATP: adenosine triphosphate and magnesium
MW: molecular weight
P-site: phosphorylation site
PDE: phosphodiesterases
PKA: protein kinase A
R-subunit: regulatory subunit of PKA
RU: resonance unit
SD: standard deviation
SPR: surface plasmon resonance
SR: sarcoplasmic reticulum

## References

[1] Shabb JB. Physiological substrates of cAMP-dependent protein kinase. Chem Rev. 2001;101(8):2381–411. 11749379 10.1021/cr000236l

[2] Taylor SS, Kornev AP. Protein kinases: evolution of dynamic regulatory proteins. Trends Biochem Sci. 2011;36(2):65–77. 10.1016/j.tibs.2010.09.006 20971646 PMC3084033

[3] Skalhegg BS, Tasken K. Specificity in the cAMP/PKA signaling pathway. Differential expression,regulation, and subcellular localization of subunits of PKA. Front Biosci. 2000;5:D678–93. 10922298 10.2741/skalhegg

[4] Taylor SS, Ilouz R, Zhang P, Kornev AP. Assembly of allosteric macromolecular switches: lessons from PKA. Nat Rev Mol Cell Bio. 2012;13(10):646–58. 22992589 10.1038/nrm3432 PMC3985763

[5] Knighton DR, Zheng JH, Ten Eyck LF, Ashford VA, Xuong NH, Taylor SS, et al Crystal structure of the catalytic subunit of cyclic adenosine monophosphate-dependent protein kinase. Science. 1991;253(5018):407–14. 1862342 10.1126/science.1862342

[6] Knighton DR, Zheng JH, Ten Eyck LF, Xuong NH, Taylor SS, Sowadski JM. Structure of a peptide inhibitor bound to the catalytic subunit of cyclic adenosine monophosphate-dependent protein kinase. Science. 1991;253(5018):414–20. 1862343 10.1126/science.1862343

[7] Bhatnagar D, Roskoski R Jr., Rosendahl MS, Leonard NJ. Adenosine cyclic 3',5'-monophosphate dependent protein kinase: a new fluorescence displacement titration technique for characterizing the nucleotide binding site on the catalytic subunit. Biochemistry. 1983;22(26):6310–7. 6318814 10.1021/bi00295a042

[8] Amieux PS, Cummings DE, Motamed K, Brandon EP, Wailes LA, Le K, et al Compensatory regulation of RIa protein levels in protein kinase A mutant mice. J Biol Chem. 1997;272:3993–8. 9020105 10.1074/jbc.272.7.3993

[9] McConnachie G, Langeberg LK, Scott JD. AKAP signaling complexes: getting to the heart of the matter. Trends Mol Med. 2006;12(7):317–23. 16809066 10.1016/j.molmed.2006.05.008

[10] Cummings DE, Brandon EP, Planas JV, Motamed K, Idzerda RL, McKnight GS. Genetically lean mice result from targeted disruption of the RII beta subunit of protein kinase A. Nature. 1996;382(6592):622–6. 8757131 10.1038/382622a0

[11] Schreyer SA, Cummings DE, McKnight GS, LeBoeuf RC. Mutation of the RIIbeta subunit of protein kinase A prevents diet-induced insulin resistance and dyslipidemia in mice. Diabetes. 2001;50(11):2555–62. 11679434 10.2337/diabetes.50.11.2555

[12] Czyzyk TA, Sikorski MA, Yang L, McKnight GS. Disruption of the RIIbeta subunit of PKA reverses the obesity syndrome of Agouti lethal yellow mice. Proc Natl Acad Sci U S A. 2008;105(1):276–81. 10.1073/pnas.0710607105 18172198 PMC2224200

[13] Erlichman J, Rosenfeld R, Rosen OM. Phosphorylation of a cyclic adenosine 3':5'-monophosphate-dependent protein kinase from bovine cardiac muscle. J Biol Chem. 1974;249(15):5000–3. 4367815

[14] Wong W, Scott JD. AKAP signalling complexes: focal points in space and time. Nat Rev Mol Cell Biol. 2004;5(12):959–70. 15573134 10.1038/nrm1527

[15] Burgers PP, Ma Y, Margarucci L, Mackey M, van der Heyden MA, Ellisman M, et al A small novel A-kinase anchoring protein (AKAP) that localizes specifically protein kinase A-regulatory subunit I (PKA-RI) to the plasma membrane. J Biol Chem. 2012;287(52):43789–97. 10.1074/jbc.M112.395970 23115245 PMC3527963

[16] Kovanich D, van der Heyden MA, Aye TT, van Veen TA, Heck AJ, Scholten A. Sphingosine kinase interacting protein is an A-kinase anchoring protein specific for type I cAMP-dependent protein kinase. Chembiochem. 2010;11(7):963–71. 10.1002/cbic.201000058 20394097

[17] Lygren B, Carlson CR, Santamaria K, Lissandron V, McSorley T, Litzenberg J, et al AKAP complex regulates Ca2+ re-uptake into heart sarcoplasmic reticulum. EMBO Rep. 2007;8(11):1061–7. 17901878 10.1038/sj.embor.7401081 PMC2247390

[18] Fischmeister R, Castro LR, Abi-Gerges A, Rochais F, Jurevicius J, Leroy J, et al Compartmentation of cyclic nucleotide signaling in the heart: the role of cyclic nucleotide phosphodiesterases. Circ Res. 2006;99(8):816–28. 17038651 10.1161/01.RES.0000246118.98832.04

[19] Zhang P, Smith-Nguyen EV, Keshwani MM, Deal MS, Kornev AP, Taylor SS. Structure and Allostery of the PKA RIIbeta Tetrameric Holoenzyme. Science. 2012;335(6069):712–6. 10.1126/science.1213979 22323819 PMC3985767

[20] Ilouz R, Bubis J, Wu J, Yim YY, Deal MS, Kornev AP, et al Localization and quaternary structure of the PKA RIbeta holoenzyme. Proc Natl Acad Sci U S A. 2012;109(31):12443–8. 10.1073/pnas.1209538109 22797896 PMC3411989

[21] Zhang P, Kornev AP, Wu J, Taylor SS. Discovery of Allostery in PKA Signaling. Biophysical Review (Accepted). 2015. 10.1007/s12551-015-0170-x PMC4469037 26097522

[22] Wu J, Brown SH, von Daake S, Taylor SS. PKA type IIalpha holoenzyme reveals a combinatorial strategy for isoform diversity. Science. 2007;318(5848):274–9. 17932298 10.1126/science.1146447 PMC4036697

[23] Kim C, Cheng CY, Saldanha SA, Taylor SS. PKA-I holoenzyme structure reveals a mechanism for cAMP-dependent activation. Cell. 2007;130(6):1032–43. 17889648 10.1016/j.cell.2007.07.018

[24] Sugden PH, Holladay LA, Reimann EM, Corbin JD. Purification and characterization of the catalytic subunit of adenosine 3':5'-cyclic monophosphate-dependent protein kinase from bovine liver. Biochem J. 1976;159(2):409–22. 187175 10.1042/bj1590409 PMC1164129

[25] Kemp BE, Graves DJ, Benjamini E, Krebs EG. Role of multiple basic residues in determining the substrate specificity of cyclic AMP-dependent protein kinase. J Biol Chem. 1977;252(14):4888–94. 194899

[26] Rangel-Aldao R, Rosen OM. Dissociation and reassociation of the phosphorylated and nonphosphorylated forms of adenosine 3':5'-monophosphate-dependent protein kinase from bovine cardiac muscle. J Biol Chem. 1976;251(11):3375–80. 179996

[27] Bastidas AC, Deal MS, Steichen JM, Guo Y, Wu J, Taylor SS. Phosphoryl transfer by protein kinase A is captured in a crystal lattice. J Am Chem Soc. 2013;135(12):4788–98. 10.1021/ja312237q 23458248 PMC3663052

[28] Grant BD, Adams JA. Pre-steady-state kinetic analysis of cAMP-dependent protein kinase using rapid quench flow techniques. Biochemistry. 1996;35(6):2022–9. 8639687 10.1021/bi952144+

[29] Manni S, Mauban JH, Ward CW, Bond M. Phosphorylation of the cAMP-dependent protein kinase (PKA) regulatory subunit modulates PKA-AKAP interaction, substrate phosphorylation, and calcium signaling in cardiac cells. J Biol Chem. 2008;283(35):24145–54. 10.1074/jbc.M802278200 18550536 PMC2527120

[30] Herberg FW, Doyle ML, Cox S, Taylor SS. Dissection of the nucleotide and metal-phosphate binding sites in cAMP-dependent protein kinase. Biochemistry. 1999;38(19):6352–60. 10320366 10.1021/bi982672w

[31] Krishnamurthy S, Moorthy BS, Liqin L, Anand GS. Dynamics of phosphodiesterase-induced cAMP dissociation from protein kinase A: capturing transient ternary complexes by HDXMS. Biochim Biophys Acta. 2013;1834(6):1215–21. 10.1016/j.bbapap.2013.02.028 23501673

[32] Dell'Acqua ML, Dodge KL, Tavalin SJ, Scott JD. Mapping the protein phosphatase-2B anchoring site on AKAP79. Binding and inhibition of phosphatase activity are mediated by residues 315–360. J Biol Chem. 2002;277(50):48796–802. 12354762 10.1074/jbc.M207833200 PMC3923414

[33] Li H, Rao A, Hogan PG. Interaction of calcineurin with substrates and targeting proteins. Trends Cell Biol. 2011;21(2):91–103. 10.1016/j.tcb.2010.09.011 21115349 PMC3244350

[34] Brown SH, Wu J, Kim C, Alberto K, Taylor SS. Novel isoform-specific interfaces revealed by PKA RIIbeta holoenzyme structures. J Mol Biol. 2009;393(5):1070–82. 10.1016/j.jmb.2009.09.014 19748511 PMC3435109

[35] Johnson DA, Akamine P, Radzio-Andzelm E, Madhusudan M, Taylor SS. Dynamics of cAMP-dependent protein kinase. Chem Rev. 2001;101(8):2243–70. 11749372 10.1021/cr000226k

