# Supplementary figures and images for: Single Turnover Autophosphorylation Cycle of the PKA RIIβ Holoenzyme
Source: PLoS Biol. 2015 Jul 9;13(7):e1002192. doi: 10.1371/journal.pbio.1002192 (PMC4497662; doi:10.1371/journal.pbio.1002192)

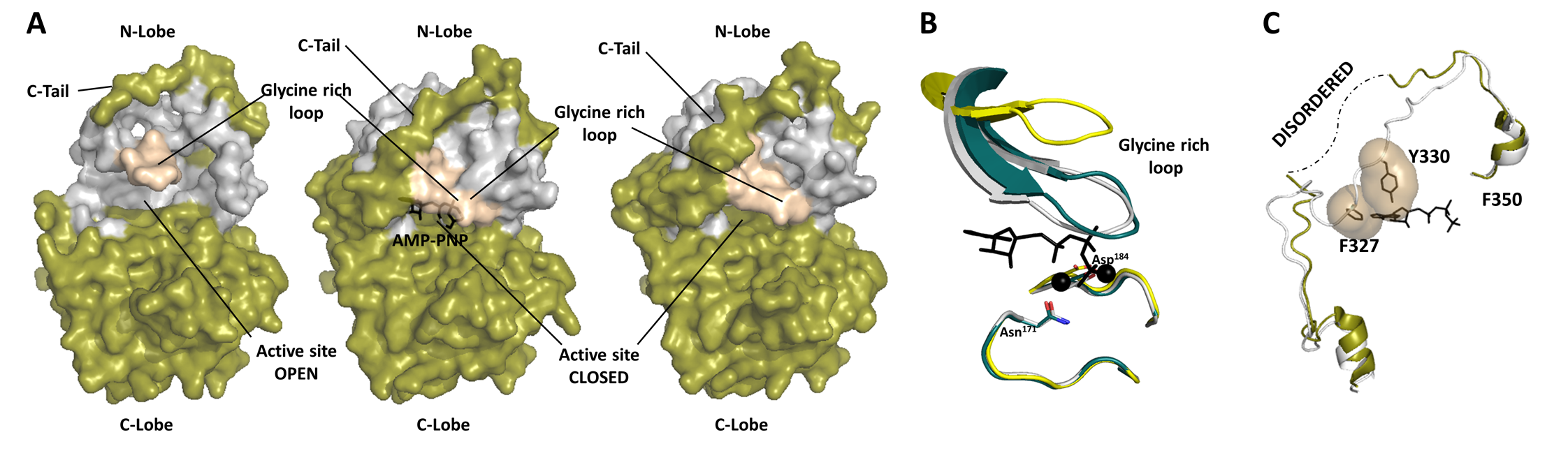

Supplement: S1 Fig — (A) The C-subunit is shown as a space-filling model with the N-lobe residues (14–121) in white and the C-lobe residues (122–350) in tan. The C-subunit conformations are termed “open” and “closed” on the basis of the relative orientations of the N- and C-lobes with respect to each other [35]. Left: The C-subunit is in a typical open conformation without nucleotide binding to the active site (in RIIα:C heterodimer [22], PDB ID: 2QVS). The glycine-rich loop (pink) is raised. The active site is open. In the open conformation in the absence of ATP, the C-tail in this region is disordered. Middle: The C-subunit is in a typical closed conformation with nucleotide binding to the active site (in RIα:C heterodimer [23], PDB ID: 2QCS). The active site is closed with nucleotide binding. The C-tail is ordered and folded over the N-lobe. Right: The C-subunit is in a closed conformation without nucleotide binding to the active site (only seen in RIIβ holoenzyme, PDB ID: 3TNP). (B) The glycine-rich loop is raised and the active site is open in the 2QVS structure (depicted in yellow), compared to the fully closed conformation observed in the 2QCS (depicted in cyan) and 3TNP structures (depicted in grey). AMP-PNP and two Mn ions from 2QCS are colored black. (C)The dynamic nature of the C-tail. In the closed conformation, the C-terminal tail (colored olive, PDB ID: 2QCS) is folded over the N-lobe in which two residues, Phe327 and Tyr300, are an essential part of the ATP binding site. In the open conformation in the absence of ATP, the C-tail (colored gray, PDB ID: 2QVS) in this region is disordered (TIF) [file pbio.1002192.s002.tif]

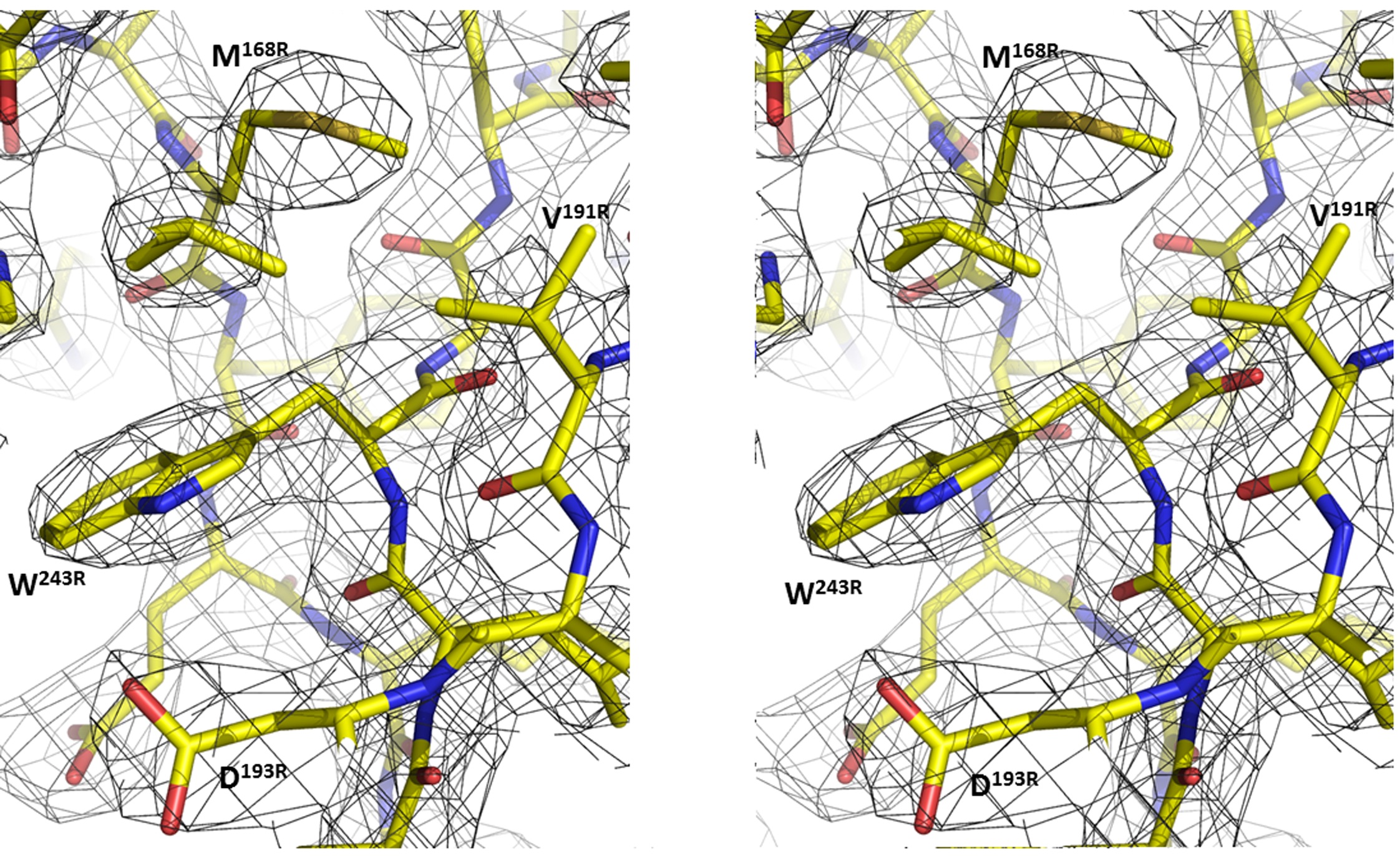

Supplement: S2 Fig — (TIF) [file pbio.1002192.s003.tif]

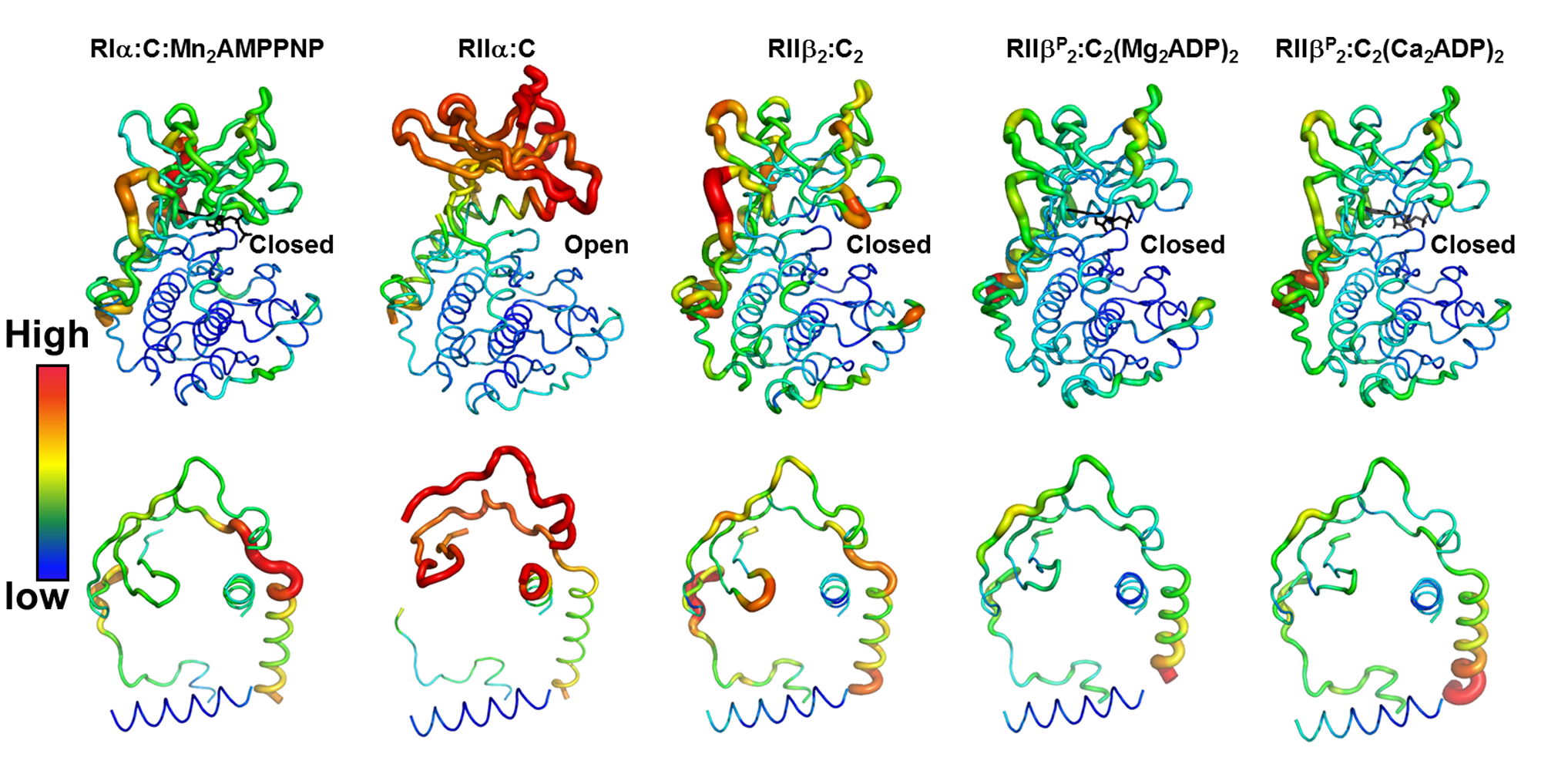

Supplement: S3 Fig — The left bottom is the scale bar for B factors. (TIF) [file pbio.1002192.s004.tif]

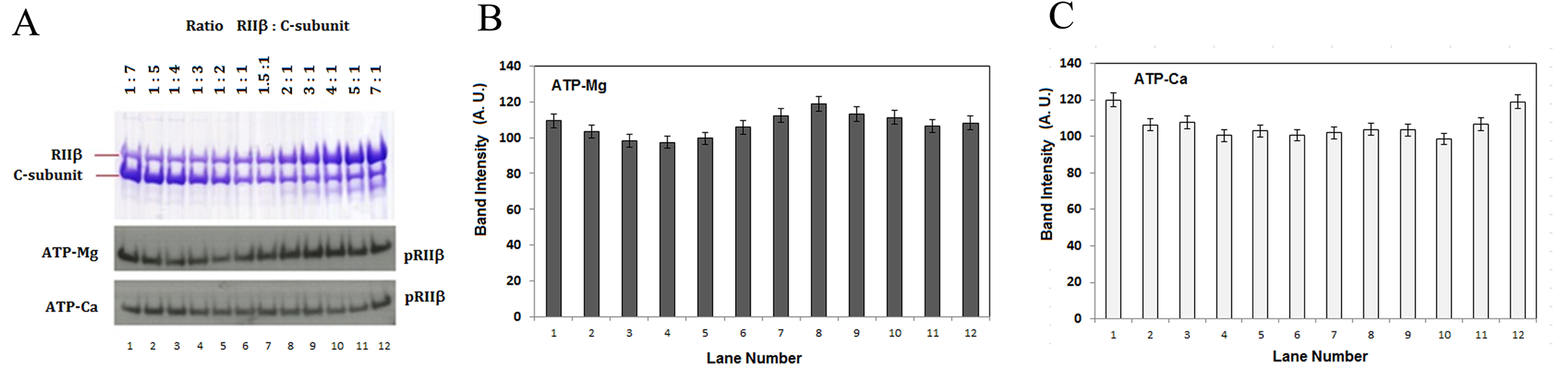

Supplement: S4 Fig — (A) Varying ratios of RIIβ were incubated with the C-subunit (in the absence of cAMP) with MgATP and CaATP. Phosphorylation of RIIβ was assessed by phosphor-Ser specific antibodies. Band intensities for the western blots were quantified using ImageJ software, as described previously [27]. (B) Band intensities in the presence of MgATP. (C) Band intensities in the presence of CaATP. The data used to make this figure can be found in S1 Data. (TIF) [file pbio.1002192.s005.tif]

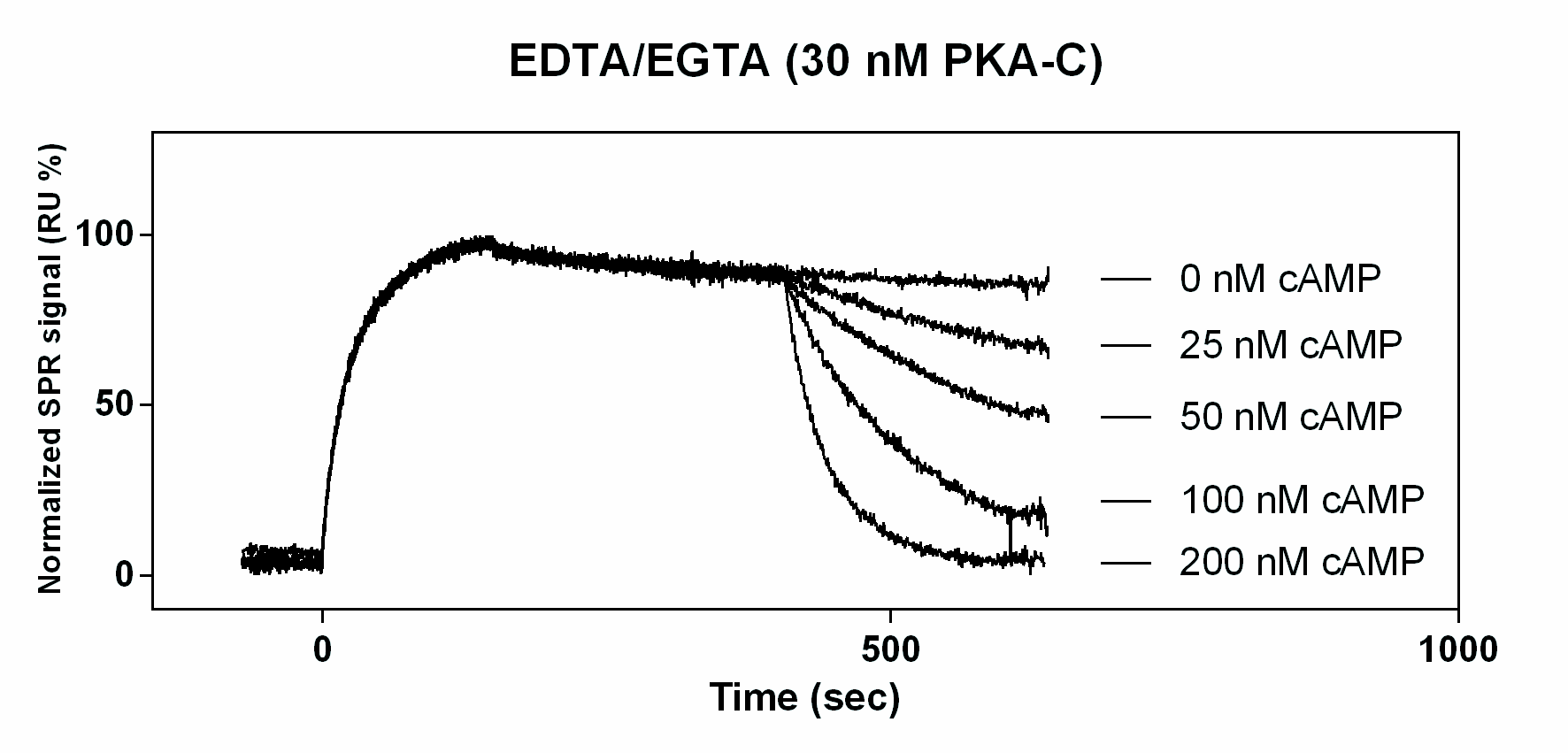

Supplement: S5 Fig — SPR was used to monitor the dissociation of the RIIβ holoenzyme (GST-RIIβ 102–416:C) induced by the injection of various concentrations of cAMP (25–200 nM). The representative plot shows holoenzyme formation with C-subunit (30 nM) and dissociation in buffer lacking ATP and metal ions (EDTA/EGTA 100 μM each). (TIF) [file pbio.1002192.s006.tif]

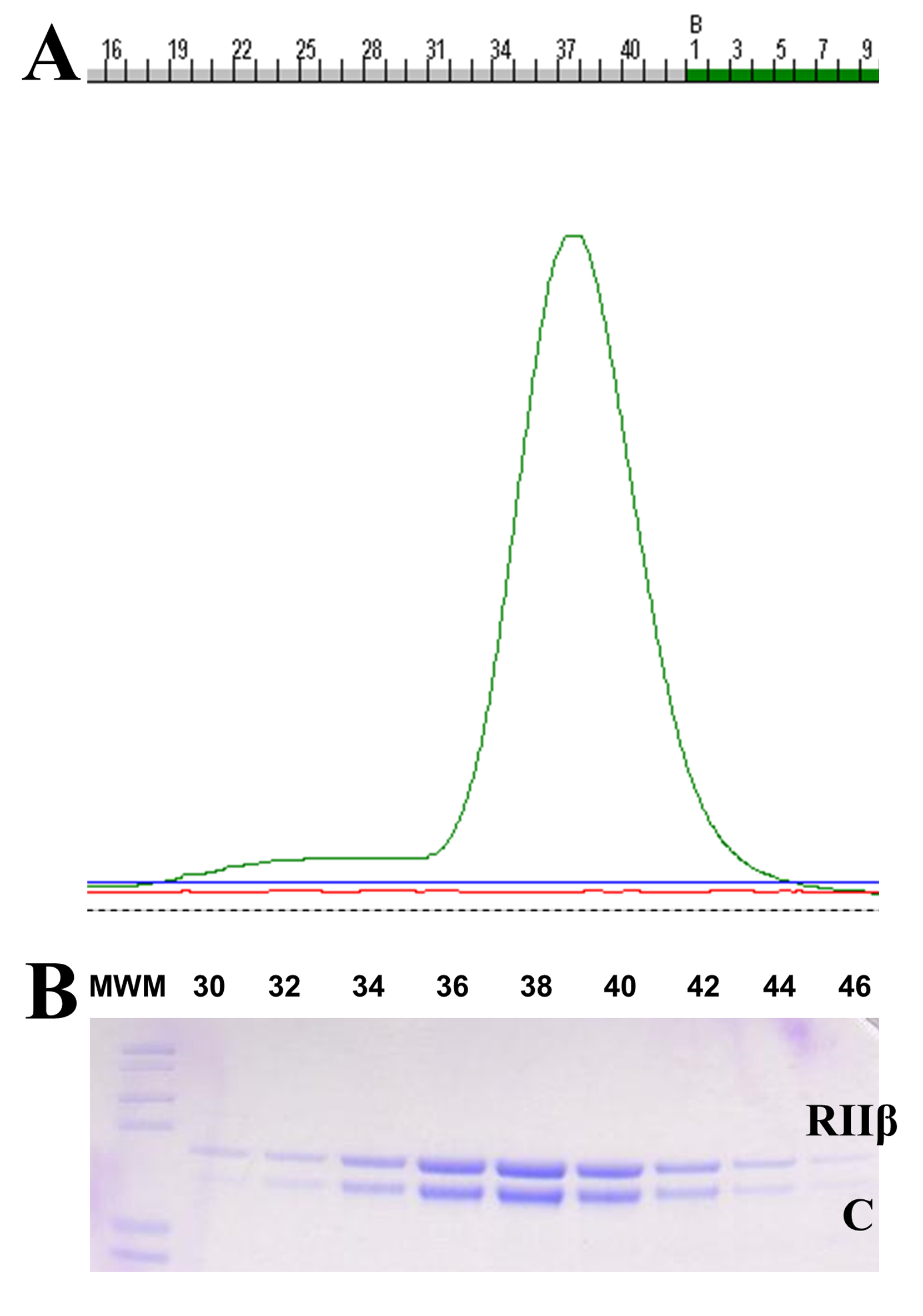

Supplement: S6 Fig — (A) Gel filtration chromatography shows that the preformed RIIβ holoenzyme does not dissociate in MgATP solution. Buffer condition: 200 mM NaCl, 20 mM MES, 1.0 mM TCEP, 5 mM MgCl2 and 1 mM ATP, pH 5.8 (B) SDS-PAGE analysis of the holoenzyme peak. (TIF) [file pbio.1002192.s007.tif]

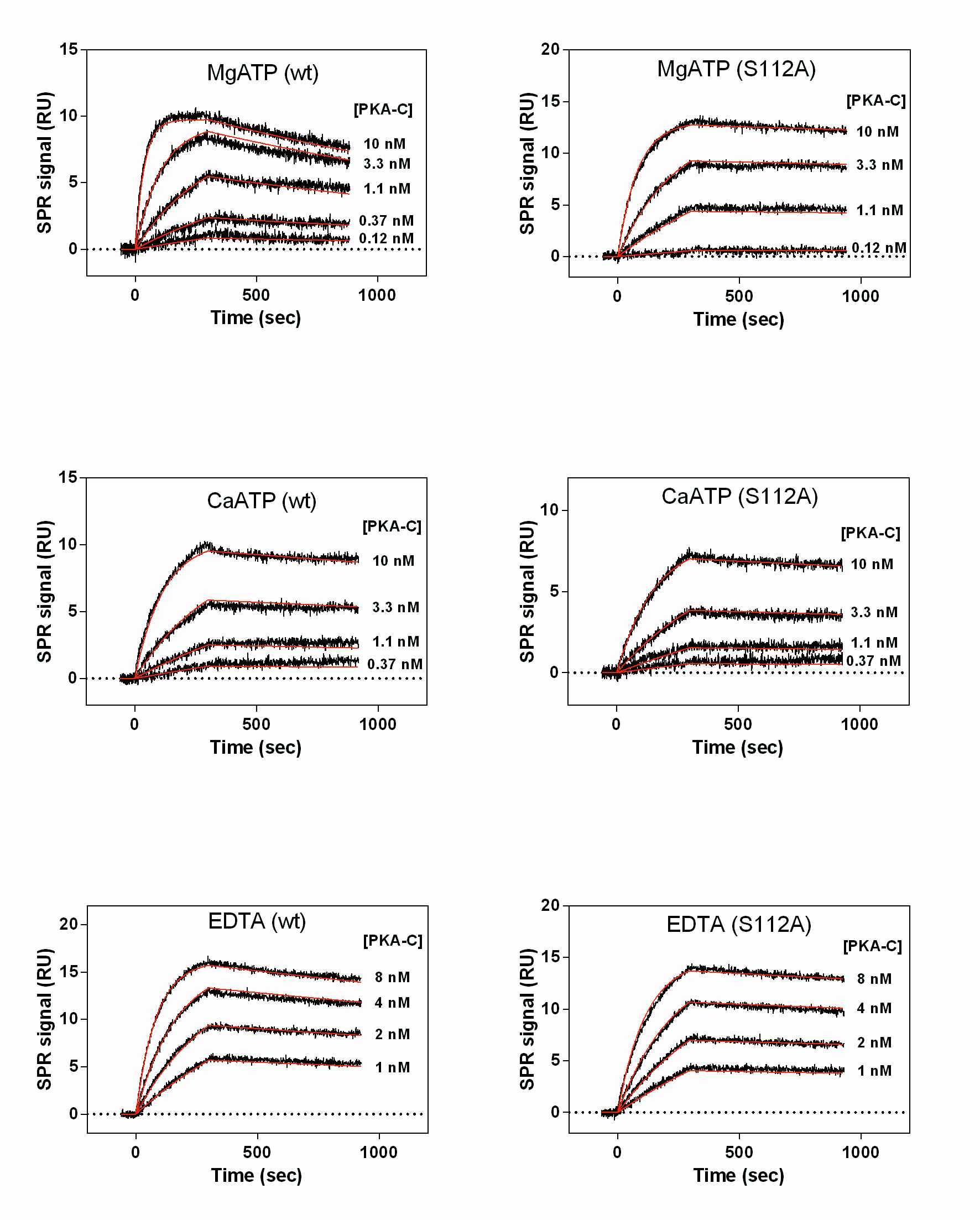

Supplement: S7 Fig — SPR was utilized to acquire binding data of holoenzyme formation for wild-type RIIβ and the P-site mutant S112A. Rate constants were determined using the Biacore T100 Evaluation Software (GE Healthcare), with global fit analysis assuming a 1:1 Langmuir binding model. (TIF) [file pbio.1002192.s008.tif]
