# Supplementary material for: Single Turnover Autophosphorylation Cycle of the PKA RIIβ Holoenzyme
Source: PLoS Biol. 2015 Jul 9;13(7):e1002192. doi: 10.1371/journal.pbio.1002192 (PMC4497662; doi:10.1371/journal.pbio.1002192)
Supplement: S1 Table — (DOCX) [file pbio.1002192.s009.docx]

**S1 Table**  **Data collection and refinement statistics**

|  | RIIβ^P^_2_: C_2_: (Ca_2_ADP)_2_ |
| --- | --- |
| **Data collection** | Advanced Light Source beamline 8.2.2 |
| Space group | C222 |
| Cell dimensions |  |
| *a*, *b*, *c* (Å) | 150.38, 213.95, 61.97 |
| α, β, γ (°) | 90, 90, 90 |
| Resolution (Å) | 2.8 |
| *R*_sym_ or *R*_merge_^a^ | 0.074 (0.49) |
| *I* / σ*I* | 23.6 (2.7) |
| Completeness (%) | 91.7 (91.8) |
| Redundancy | 3.5 |
|  |  |
| **Refinement** |  |
| Resolution (Å) | 40.0-2.8 |
| No. reflections | 23056 |
| *R*_work_ / *R*_free_ ^b^ | 23.2/27.9 |
| No. atoms |  |
| Protein | 4914 |
| Ligand/ion | 1 |
| Water | 42 |
| *B*-factors |  |
| Protein | 55.21 |
| Ligand/ion | 44.30 |
| Water | 58.97 |
| R.m.s. deviations |  |
| Bond lengths (Å) | 0.014 |
| Bond angles (°) | 1.48 |
| Ramachandran angles(%)^c^ |  |
| Favored regions | 90.6 |
| Allowed regions | 100 |

Data collection was performed at ALS laboratory in Berkeley, CA on beamline 8.2.2. ^a^Values in parenthesis correspond to the highest resolution shell. ^b^5% of the data was excluded from the refinement to calculate the Rfree. ^c^Ramachandran plot quality as defined in Procheck.
